# Impact of post-transfusion hemoglobin levels on survival in critically Ill patients: a machine learning–based causal inference analysis

**DOI:** 10.1038/s41598-026-50363-y

**Published:** 2026-04-25

**Authors:** Min Woo Kang, Soojeong Yun, Seung Min Song, Ji Eun Kim, Hyo Jin Kim, Eun Jung Cho, Young Joo Kwon, Shin Young Ahn

**Affiliations:** 1 Department of Internal Medicine, Korea University Guro Hospital, Seoul, Korea; 2 Department of Internal Medicine, Korea University College of Medicine, Seoul, Korea; 3 Department of Internal Medicine Hospital Medical Center, Seoul National University Bundang Hospital, 82, Gumi-ro 173 Beon-gil, Bundang-gu, Seongnam-si, Gyeonggi-do 13620 Korea

**Keywords:** Red blood cell transfusion, Hemoglobin targets, Intensive care unit, Causal inference, Machine learning, Biomarkers, Cardiology, Diseases, Medical research, Risk factors

## Abstract

Restrictive red blood cell transfusion strategies (hemoglobin trigger 7.0–8.0 g/dL) are well established, yet existing evidence addresses primarily when to initiate transfusion. The optimal achieved post-transfusion hemoglobin level in critically ill patients with severe anemia (pre-transfusion hemoglobin ≤ 8.0 g/dL) remains unknown. We analyzed 3,710 intensive care unit admissions in the Medical Information Mart for Intensive Care IV (MIMIC-IV; 3,339 training, 371 test) and externally validated findings in 4,719 admissions from the eICU Collaborative Research Database. Eligible subjects received red blood cells within 48 h of ICU admission, had a pre-transfusion hemoglobin ≤ 8.0 g/dL, and had no additional transfusions between 48 h and seven days. Using a double machine learning causal forest, we estimated treatment effects for post-transfusion hemoglobin targets from 8.0 to 14.0 g/dL relative to 8.0 g/dL, identified the target with the lowest predicted in-hospital mortality, evaluated subgroups, and modeled predictors of targets below 10.0 g/dL. Dose–response curves were U-shaped in all datasets. The estimated optimal hemoglobin was 11.0 g/dL in the training set, 10.9 g/dL in the test set, and 11.0 g/dL in external validation. Estimated optimal levels near 11.0 g/dL were consistent across chronic kidney disease, end-stage kidney disease, myocardial infarction, congestive heart failure, hypertension, and diabetes. Lower baseline hemoglobin and lower estimated glomerular filtration rate were associated with lower individualized optimal levels. Indicators of inflammation or hemodynamic instability, including hypotension, fever, leukocytosis, hyperlactatemia, acidosis, and vasopressor requirements, were associated with estimated optimal achieved post-transfusion hemoglobin levels below 10.0 g/dL. Among ICU subjects transfused under a restrictive trigger (hemoglobin ≤ 8.0 g/dL), an achieved post-transfusion hemoglobin around 11.0 g/dL was associated with the lowest predicted in-hospital mortality.

**Supplementary Information:**

The online version contains supplementary material available at 10.1038/s41598-026-50363-y.

## Introduction

Anemia is common in critically ill patients, and red blood cell (RBC) transfusion is frequently employed to correct it^1,2^. Early landmark randomized trials have generally shown that a restrictive strategy (trigger at hemoglobin < 7.0–8.0 g/dL) is noninferior to a liberal approach (trigger at hemoglobin < 9.0–10.0 g/dL) for mortality and major morbidity^3,4^. Subsequent evidence syntheses have reinforced these findings, demonstrating no consistent advantage of liberal over restrictive thresholds^5–8^. Reflecting this body of work, current practice guidelines advise restrictive transfusion practices in hemodynamically stable intensive care unit (ICU) patients^9^.

Despite robust evidence regarding when to start transfusion, there remains a paucity of evidence on the optimal hemoglobin concentration to maintain after transfusion, particularly in patients presenting with severe anemia (pre-transfusion hemoglobin ≤ 8.0 g/dL). Importantly, the achieved post-transfusion hemoglobin level—the concentration actually attained after red blood cell (RBC) administration—is distinct from the transfusion initiation threshold. In routine ICU practice, transfusion is guided by a trigger rather than a predefined post-transfusion goal; standard recommendations advise transfusing one unit at a time and reassessing, so the resulting hemoglobin depends on individual response, ongoing blood loss, hemodilution, and the number of units administered. Moreover, red blood cell units themselves vary in hemoglobin content and volume depending on processing methods and storage conditions, adding a further source of variability in the post-transfusion hemoglobin response^10^. Consequently, although substantial evidence informs when to initiate transfusion, clinicians have little guidance on what post-transfusion hemoglobin level should be achieved to optimize patient outcomes. This gap has important clinical implications: insufficient correction may perpetuate tissue hypoxia, whereas excessive transfusion can increase the risk of complications such as transfusion-related acute lung injury, volume overload, and immunomodulation^11–13^.

Advancements in machine learning and causal inference methodologies offer novel avenues to address complex clinical questions using observational data^14^. Machine learning-based causal inference models enable the estimation of individualized treatment effects while accounting for confounding variables^15^. These approaches have been successfully applied in critical care research to evaluate interventions where RCTs are impractical or pose ethical challenges^16^.

In this study, we employed a machine learning-based causal inference framework to determine the optimal achieved post-transfusion hemoglobin level associated with the lowest in-hospital mortality among ICU patients who received RBC transfusions within 48 h of admission and had pre-transfusion hemoglobin levels ≤ 8.0 g/dL. We leveraged the Medical Information Mart for Intensive Care (MIMIC)-IV database and the Electronic Intensive Care Unit Collaborative Research Database (eICU)—large, rigorously validated critical-care datasets that have underpinned numerous prior investigations. By concentrating on post-transfusion hemoglobin targets rather than initiation thresholds, our study addresses a vital yet underexplored dimension of transfusion management in the ICU.

## Methods

### Study population

We leveraged version 3.1 of the publicly available MIMIC-IV database for model development and internal validation. MIMIC-IV comprises de-identified, longitudinal ICU data from Beth Israel Deaconess Medical Center collected between 2008 and 2022, encompassing over 94,000 patient admissions and detailed demographic, physiologic, laboratory, medication, procedural, and diagnostic records^17^. For external validation, we used version 2.0 of the eICU Collaborative Research Database, a multi-center dataset that aggregates more than 200,000 ICU admissions from over 200 hospitals across the United States during 2014–2015, representing approximately 139,000 unique patients with analogous clinical variables^18^. The geographic diversity and multi-center design of eICU enhance the generalizability of our findings.

Inclusion was restricted to ICU admissions that received an RBC transfusion within the first 48 h of admission and had a pre-transfusion nadir hemoglobin of ≤ 8.0 g/dL, reflecting a restrictive initiation threshold (Supplementary Figure S1). To ensure that the recorded post-transfusion hemoglobin represented the final target level, admissions receiving any additional transfusions between 48 h and seven days after ICU admission were excluded. We excluded admissions involving cardiopulmonary bypass during cardiac surgery, given evidence that intraoperative RBC transfusion in this setting is associated with a heightened risk of acute kidney injury^19^. This restriction was used to emulate a single early transfusion episode and to define the post-transfusion hemoglobin as a stable exposure (i.e., not subsequently altered by additional transfusions). The resulting development data was then randomly allocated in a 9:1 ratio to the training and test sets.

### Treatment, outcome, and feature variables

This study incorporated a comprehensive set of variables, including demographic characteristics, laboratory results, comorbidities, patient status, and treatment details. Variables with more than 30% missing data in the development data were excluded to ensure robustness. The remaining missing values were imputed using Multivariate Imputation by Chained Equations (MICE)^20,21^. Excluded variables due to excessive missing data included total protein, C-reactive protein, height, and albumin, as detailed in Supplementary Table S1. For the external validation data, the proportion of missing data in the included variables is presented in Supplementary Table S2, and the missing values were similarly imputed using the MICE method.

The treatment variable was defined as the hemoglobin concentration measured within 48 h after the final RBC transfusion, selecting the value closest to the end of transfusion to represent the achieved post-transfusion hemoglobin level. The primary outcome was in-hospital mortality. We adjusted for a comprehensive set of baseline covariates encompassing demographic characteristics, initial vital signs, respiratory support parameters, laboratory measurements, vasoactive and inotropic agent infusion rates, and major comorbidities (the complete list of variables and their definitions is provided in Supplementary Table S3). Baseline covariates were intentionally restricted to the first 24 h of ICU admission to capture the pre-transfusion physiological state and minimize the risk of adjusting for post-treatment variables, whereas the 48-hour transfusion window was chosen to ensure adequate sample size while still reflecting early ICU management. For each variable, we selected the clinically worst value observed during the first 24 h of ICU admission, defined a priori according to the expected direction of greater physiologic derangement. Specifically, maximum values were used for creatinine, FiO₂, temperature, WBC count, serum calcium, anion gap, BUN, potassium, AST, ALT, total bilirubin, PT–INR, aPTT, lactate, and vasoactive infusions, whereas minimum values were used for blood pressures, initial hemoglobin, eGFR, SpO₂, platelet count, pH, bicarbonate, sodium, and chloride. To minimize the influence of measurement artifacts, physiologically implausible values were excluded during data extraction and preprocessing; thus, these summaries were intended to capture early illness severity rather than erroneous outlier measurements. To make explicit the assumed causal structure underlying the analysis, particularly the temporal relationship among transfusion, achieved post-transfusion hemoglobin, early clinical covariates, and mortality, we constructed a directed acyclic graph (Supplementary Figure S2).

### Development and hyperparameter optimization of the double machine learning causal forest model

We employed a double machine learning–based causal forest estimator to quantify individualized treatment effects of post-transfusion hemoglobin on in-hospital mortality. To capture potential nonlinear associations, the continuous hemoglobin treatment variable was expanded via a polynomial transformation, with the degree of that transformation treated as one of the hyperparameters. Under this framework, one random forest–based learner estimated the outcome model, while a second random forest–based learner estimated the treatment model.

Hyperparameter selection proceeded via grid search over four parameters: the number of decision trees (500, 1,000, 1,500), maximum tree depth (5, 10, 15), minimum samples per leaf (3, 5, 10), and degree of the polynomial transformation (3, 5, 7). We conducted tenfold cross-validation on the MIMIC-IV training cohort, choosing the parameter combination that minimized a bias-corrected mean squared error on held-out folds.

With the optimal settings determined, the final causal forest estimator was retrained on the entire development data. We then characterized the dose–response relationship by estimating the average treatment effect (ATE) for hemoglobin targets from 8.0 to 14.0 g/dL, sampled at 100 equally spaced values. For each candidate target, the individual treatment effect was defined as the difference in predicted outcome between setting hemoglobin to the candidate level versus a baseline target of 8.0 g/dL, which represents the restrictive transfusion initiation threshold and conventional post-transfusion goal^5,8,9,22^. These individual effects were averaged across all patients to yield the ATE. The hemoglobin value associated with the lowest ATE was designated the estimated optimal level, and treatment effect curves were generated for the training, test, and external validation data. We selected the double machine learning causal forest because the exposure of interest is continuous (post-transfusion hemoglobin target) and clinically plausible effects are nonlinear and heterogeneous across admissions. In contrast, classical propensity score matching is typically formulated for binary treatments and would require ad hoc discretization of hemoglobin targets, which can reduce information and obscure dose–response patterns. DML further reduces bias from flexible nuisance modeling through orthogonalization, supporting stable estimation in high-dimensional confounding settings.

### Model evaluation

Model performance was assessed in two complementary domains. First, we quantified the precision of heterogeneous treatment effect estimation using nearest-neighbor Precision in Estimating Heterogeneous Effects (NN-PEHE)^23,24^. The continuous post-transfusion hemoglobin variable was partitioned into nine half-unit intervals (8.0–8.5, 8.5–9.0, …, 12.5–13.0 g/dL). For each patient and each interval, we identified a nearest neighbor from every other interval by matching on baseline covariates; the observed difference in outcomes between matched pairs constituted the empirical individualized treatment effect. Our causal forest estimator then predicted treatment effects at each interval midpoint under identical covariate profiles. Squared differences between observed and predicted effects were averaged and square-rooted to yield NN-PEHE, calculated separately in the test and external validation data, with lower values indicating greater precision. Second, we evaluated the predictive accuracy and calibration of the outcome submodel. Predicted probabilities were averaged across cross-fitted outcome learners to form an ensemble estimate for each observation. We then computed the area under the receiver operating characteristic curve (AUROC) to assess discrimination, classification accuracy using a 0.5 probability threshold, and the Brier score to quantify overall probabilistic error.

### Subgroup analysis for optimal hemoglobin targets

To determine whether optimal transfusion targets varied across clinically relevant populations, we stratified patients by the presence or absence of CKD, ESKD, MI, CHF, hypertension, and diabetes, as well as by initial hemoglobin strata. Within each subgroup, we estimated ATE curves over hemoglobin targets from 8.0 to 14.0 g/dL and defined the nadir of each curve as the subgroup’s optimal hemoglobin. At the individual level, each patient’s optimal hemoglobin target was identified as the value between 8.0 and 14.0 g/dL that minimized the individual treatment effect, defined as the predicted outcome difference between that candidate level and a base hemoglobin of 8.0 g/dL. We then examined associations between individualized targets and clinical covariates, using two-sample t-tests for binary comorbidities and simple linear regression for continuous predictors (initial hemoglobin and eGFR), and reported R² to indicate the variance explained.

### Multivariable logistic regression analysis of predictors for lower optimal hemoglobin targets

A binary outcome was defined to indicate whether each patient’s individualized optimal hemoglobin target was < 10.0 g/dL. We then fitted multivariable logistic regression models using key clinical covariates as independent variables to identify patient characteristics associated with an individualized optimal hemoglobin < 10.0 g/dL. Continuous predictors were standardized prior to analysis, and all effect estimates are reported as standardized odds ratios with 95% confidence intervals. All analyses were conducted in Python 3.11.7 using the econML library for causal inference and scikit-learn for model fitting.

## Results

### Study population and baseline characteristics

**Fig. 1:**
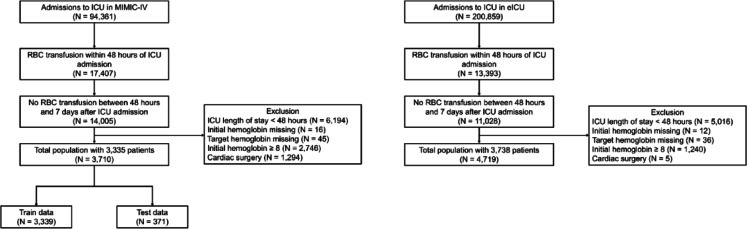
Flow diagram of the development and external validation cohort. ICU, intensive care unit; MIMIC, Medical Information Mart for Intensive Care.

In the MIMIC-IV data, 94,361 ICU admissions were initially identified (Fig. 1). Among these, 17,407 admissions received an RBC transfusion within the first 48 h, of which 14,005 had no subsequent transfusion between 48 h and 7 days. Applying exclusion criteria—ICU length of stay < 48 h (*n* = 6,194), missing initial hemoglobin (*n* = 16), missing achieved post-transfusion hemoglobin (*n* = 45), pre-transfusion hemoglobin ≥ 8.0 g/dL (*n* = 2,746), and cardiac surgery admissions (*n* = 1,294)—yielded 3,710 admissions for analysis. These were randomly allocated into train (*n* = 3,339) and test data (*n* = 371). Most baseline characteristics were comparable between the two cohorts, with no statistically significant differences observed (Supplementary Table S3). In the eICU Collaborative Research Database, 200,859 ICU admissions were evaluated. Of these, 13,393 admissions received an RBC transfusion within 48 h, and 11,028 had no further transfusions through day 7. The same exclusion criteria excluded 8,674 admissions, leaving 4,719 admissions for external validation.

In the MIMIC-IV data, mean age was 63.7 ± 15.5 years, and 52.5% of admissions were male (Table 1). Mean pre-transfusion hemoglobin was 6.8 ± 0.8 g/dL, and mean achieved post-transfusion hemoglobin was 9.0 ± 1.3 g/dL. In-hospital mortality occurred in 669 admissions (18.0%). Given the possibility of a non-normal distribution of hemoglobin values, median values are also provided: the median pre- and post-transfusion hemoglobin levels were 6.9 g/dL [6.4–7.4] and 8.7 g/dL [8.0–9.7], respectively. In the eICU data, mean age was 64.9 ± 15.2 years, with 51.7% male. Mean pre-transfusion hemoglobin was 6.6 ± 0.9 g/dL, and mean achieved post-transfusion hemoglobin was 8.9 ± 1.2 g/dL. The corresponding median pre- and post-transfusion hemoglobin levels were 6.8 g/dL [6.2–7.3] and 8.8 g/dL [8.1–9.6], respectively. In-hospital mortality was observed in 570 admissions (12.1%).

**Table 1:** Baseline characteristics.

Variable	MIMIC-IV			eICU		
	Total population (*N* = 3710)	No in-hospital mortality (*N* = 3041)	In-hospital mortality (*N* = 669)	Total population (*N* = 4719)	No in-hospital mortality (*N* = 4149)	In-hospital mortality (*N* = 570)
**Age (years)**	63.7 ± 15.5	63.2 ± 15.6	65.7 ± 14.8	64.9 ± 15.2	64.7 ± 15.2	66.5 ± 14.7
**Weight (kg)**	77.6 ± 19.9	77.6 ± 20.1	77.5 ± 19.0	81.0 ± 24.4	81.2 ± 24.3	79.7 ± 25.3
**Achieved post-transfusion hemoglobin (g/dL)**	9.0 ± 1.3	9.0 ± 1.3	8.9 ± 1.3	8.9 ± 1.2	8.9 ± 1.2	9.2 ± 1.4
**Length of ICU stay (hours)**	126 ± 136	119 ± 118	161 ± 195	120 ± 113	113 ± 96	169 ± 189
**Heart rate (beats/min)**	114 ± 22	113 ± 21	118 ± 22	114 ± 22	113 ± 22	118 ± 24
**SBP (mmHg)**	83 ± 15	84 ± 15	78 ± 16	79 ± 13	80 ± 13	77 ± 15
**DBP (mmHg)**	40 ± 10	41 ± 10	39 ± 10	39 ± 8	39 ± 8	38 ± 7
**Temperature (°C)**	37.6 ± 0.8	37.6 ± 0.7	37.5 ± 0.9	37.8 ± 0.5	37.8 ± 0.5	37.7 ± 0.6
**SpO₂ (%)**	89.7 ± 7.4	90.0 ± 6.8	88.0 ± 9.3	85.4 ± 11.8	85.8 ± 11.3	82.1 ± 14.7
**Average SBP (mmHg)**	114 ± 15	115 ± 15	111 ± 14	116 ± 12	117 ± 12	112 ± 14
**Average DBP (mmHg)**	60 ± 10	60 ± 10	58 ± 10	55 ± 8	56 ± 8	53 ± 8
**FiO₂ (%)**	54.3 ± 30.9	54.6 ± 30.5	53.0 ± 32.8	23.0 ± 11.3	22.8 ± 10.9	24.3 ± 14.1
**WBC (10³/µL)**	15.7 ± 15.7	15.4 ± 16.3	16.8 ± 12.7	15.9 ± 12.5	15.6 ± 12.0	18.0 ± 15.8
**Initial hemoglobin (g/dL)**	6.8 ± 0.8	6.8 ± 0.8	6.8 ± 0.8	6.6 ± 0.9	6.7 ± 0.9	6.6 ± 0.9
**Platelet (10³/µL)**	166 ± 126	172 ± 127	138 ± 113	231 ± 140	235 ± 139	203 ± 138
**ALT (U/L)**	139 ± 485	125 ± 392	203 ± 778	127 ± 469	101 ± 385	316 ± 836
**AST (U/L)**	256 ± 1042	215 ± 738	446 ± 1874	271 ± 1205	200 ± 883	786 ± 2463
**Total bilirubin (mg/dL)**	2.9 ± 6.0	2.5 ± 5.2	5.0 ± 8.7	1.7 ± 3.3	1.5 ± 2.6	3.4 ± 6.1
**Calcium (mg/dL)**	8.6 ± 1.1	8.5 ± 1.1	8.7 ± 1.2	8.5 ± 0.9	8.5 ± 0.8	8.5 ± 1.1
**pH**	7.32 ± 0.11	7.32 ± 0.10	7.28 ± 0.13	7.32 ± 0.10	7.33 ± 0.09	7.26 ± 0.13
**Bicarbonate (mmol/L)**	20.2 ± 5.1	20.5 ± 4.9	19.0 ± 5.5	20.4 ± 5.8	20.7 ± 5.3	18.3 ± 8.3
**Anion gap (mmol/L)**	17.1 ± 5.3	16.7 ± 5.1	18.8 ± 5.8	13.6 ± 5.7	13.2 ± 5.5	16.5 ± 6.8
**BUN (mg/dL)**	41 ± 30	40 ± 29	47 ± 33	41 ± 30	40 ± 30	50 ± 31
**Creatinine (mg/dL)**	2.1 ± 2.2	2.1 ± 2.3	2.2 ± 1.7	2.2 ± 2.3	2.1 ± 2.4	2.5 ± 1.9
**Sodium (mmol/L)**	136 ± 6	136 ± 6	136 ± 6	136 ± 5	136 ± 5	136 ± 6
**Potassium (mmol/L)**	4.7 ± 0.8	4.7 ± 0.8	4.7 ± 0.9	4.7 ± 0.8	4.7 ± 0.8	4.9 ± 1.0
**Chloride (mmol/L)**	101 ± 7	101 ± 7	101 ± 8	102 ± 7	102 ± 7	101 ± 8
**PT INR**	1.9 ± 1.2	1.8 ± 1.1	2.2 ± 1.2	1.9 ± 1.6	1.9 ± 1.5	2.4 ± 1.7
**aPTT (sec)**	51.0 ± 33.9	49.4 ± 33.1	58.6 ± 36.6	43.6 ± 22.4	42.6 ± 21.8	51.2 ± 25.2
**Lactate (mmol/L)**	3.1 ± 2.7	2.9 ± 2.4	4.1 ± 3.7	3.0 ± 3.0	2.7 ± 2.6	4.8 ± 4.7
**Baseline creatinine (mg/dL)**	1.7 ± 2.0	1.7 ± 2.1	1.6 ± 1.4	1.9 ± 2.2	1.9 ± 2.2	2.1 ± 1.8
**eGFR (mL/min/1.73 m²)**	55 ± 35	57 ± 36	46 ± 31	53 ± 34	55 ± 34	41 ± 29
**Baseline eGFR (mL/min/1.73 m²)**	67 ± 38	68 ± 38	63 ± 37	60 ± 35	61 ± 35	51 ± 33
**Norepinephrine (mcg/kg/min)**	0.05 ± 0.14	0.04 ± 0.14	0.08 ± 0.14	0.01 ± 0.22	0.01 ± 0.16	0.06 ± 0.47
**Dopamine (mcg/kg/min)**	0.18 ± 1.42	0.14 ± 1.22	0.36 ± 2.07	0.08 ± 0.75	0.06 ± 0.63	0.20 ± 1.30
**Epinephrine (mcg/kg/min)**	0.00 ± 0.05	0.00 ± 0.03	0.02 ± 0.11	0.03 ± 0.49	0.01 ± 0.32	0.16 ± 1.12
**Vasopressin (units/hr)**	0.27 ± 0.83	0.18 ± 0.62	0.70 ± 1.37	0.29 ± 1.21	0.19 ± 0.98	1.01 ± 2.15
**Dobutamine (mcg/kg/min)**	0.05 ± 0.49	0.04 ± 0.44	0.11 ± 0.66	0.08 ± 0.70	0.07 ± 0.66	0.14 ± 0.94
**Male**	1947 (52.5%)	1589 (52.3%)	358 (53.5%)	2441 (51.7%)	2129 (51.3%)	312 (54.7%)
**RRT within 48 h**	88 (2.4%)	49 (1.6%)	39 (5.8%)	275 (5.8%)	220 (5.3%)	55 (9.6%)
**Mechanical ventilation**	2058 (55.5%)	1722 (56.6%)	336 (50.2%)	1837 (38.9%)	1509 (36.4%)	328 (57.5%)
**Chronic kidney disease**	862 (23.2%)	688 (22.6%)	174 (26.0%)	607 (12.9%)	511 (12.3%)	96 (16.8%)
**End-stage kidney disease**	309 (8.3%)	259 (8.5%)	50 (7.5%)	313 (6.6%)	277 (6.7%)	36 (6.3%)
**Myocardial infarction**	656 (17.7%)	519 (17.1%)	137 (20.5%)	495 (10.5%)	428 (10.3%)	67 (11.8%)
**Congestive heart failure**	1187 (32.0%)	949 (31.2%)	238 (35.6%)	820 (17.4%)	724 (17.4%)	96 (16.8%)
**Peripheral vascular disease**	579 (15.6%)	462 (15.2%)	117 (17.5%)	323 (6.8%)	274 (6.6%)	49 (8.6%)
**Cerebrovascular disease**	453 (12.2%)	352 (11.6%)	101 (15.1%)	501 (10.6%)	433 (10.4%)	68 (11.9%)
**Chronic liver disease**	987 (26.6%)	744 (24.5%)	243 (36.3%)	367 (7.8%)	307 (7.4%)	60 (10.5%)
**Diabetes**	1235 (33.3%)	1025 (33.7%)	210 (31.4%)	1436 (30.4%)	1278 (30.8%)	158 (27.7%)
**Hypertension**	1609 (43.4%)	1345 (44.2%)	264 (39.5%)	2423 (51.3%)	2131 (51.4%)	292 (51.2%)
**Non-cardiac surgery**	543 (14.6%)	467 (15.4%)	76 (11.4%)	9 (0.2%)	9 (0.2%)	0 (0.0%)

MIMIC, Medical Information Mart for Intensive Care; eICU, electronic intensive care unit; ICU, intensive care unit; SBP, systolic blood pressure; DBP diastolic blood pressure; SpO₂, saturation of peripheral oxygen; FiO₂, fraction of inspired oxygen; WBC, white blood cell; ALT, alanine aminotransferase; AST, aspartate aminotransferase; BUN, blood urea nitrogen; PT INR, prothrombin time international normalized ratio; aPTT, activated partial thromboplastin time; eGFR, estimated glomerular filtration rate; RRT, renal replacement therapy.

### Model development and performance evaluation

Ten-fold cross-validation identified an optimal causal forest model using 1,000 trees, a maximum depth of 5, minimum leaf samples of 3, and a third-degree polynomial transformation of the treatment variable, which yielded the lowest bias-corrected mean squared error. Under this configuration, precision in estimating heterogeneous treatment effects—as quantified by NN-PEHE—was 0.5134 in the test data and 0.4752 in the external validation data (Supplementary Table S4). The ensemble outcome predictor, formed by averaging probability estimates across cross-fitted learners, achieved an AUROC of 0.7667, accuracy of 0.8329, and Brier score of 0.1249 in the MIMIC-IV test data; in the external validation data, it achieved an AUROC of 0.7655, accuracy of 0.8813, and Brier score of 0.0954.

### Average treatment effect curves and optimal hemoglobin targets

**Fig. 2:**
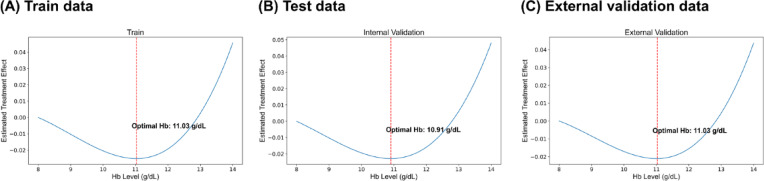
Average treatment effect curves for post-transfusion hemoglobin targets on in-hospital mortality. Hb, hemoglobin; (**A**), Train data. (**B**), Test data. (**C**), External validation data.

ATE curves in the training, test, and external validation data all exhibited a U-shaped relationship with post‐transfusion hemoglobin (Fig. 2). The nadir of each curve, indicating the optimal hemoglobin target, was 11.0 g/dL in the training data, 10.9 g/dL in the test data, and 11.0 g/dL in the external validation data. We additionally generated supplementary curves re-centered on the individualized optimal hemoglobin level associated with the lowest predicted mortality, together with 95% confidence intervals (Supplementary Figure S3).

### Subgroup analyses and clinical correlates of individualized optimal hemoglobin targets

Subgroup analyses demonstrated notable consistency in the estimated optimal achieved post-transfusion hemoglobin level—approximately 11.0 g/dL—regardless of CKD, ESKD, MI, CHF, hypertension, or diabetes across the training, internal validation, and external validation data (Supplementary Figure S4). Supplementary Figure S4c further illustrates that lower initial hemoglobin corresponded to lower individualized optimal hemoglobin targets.

**Fig. 3:**
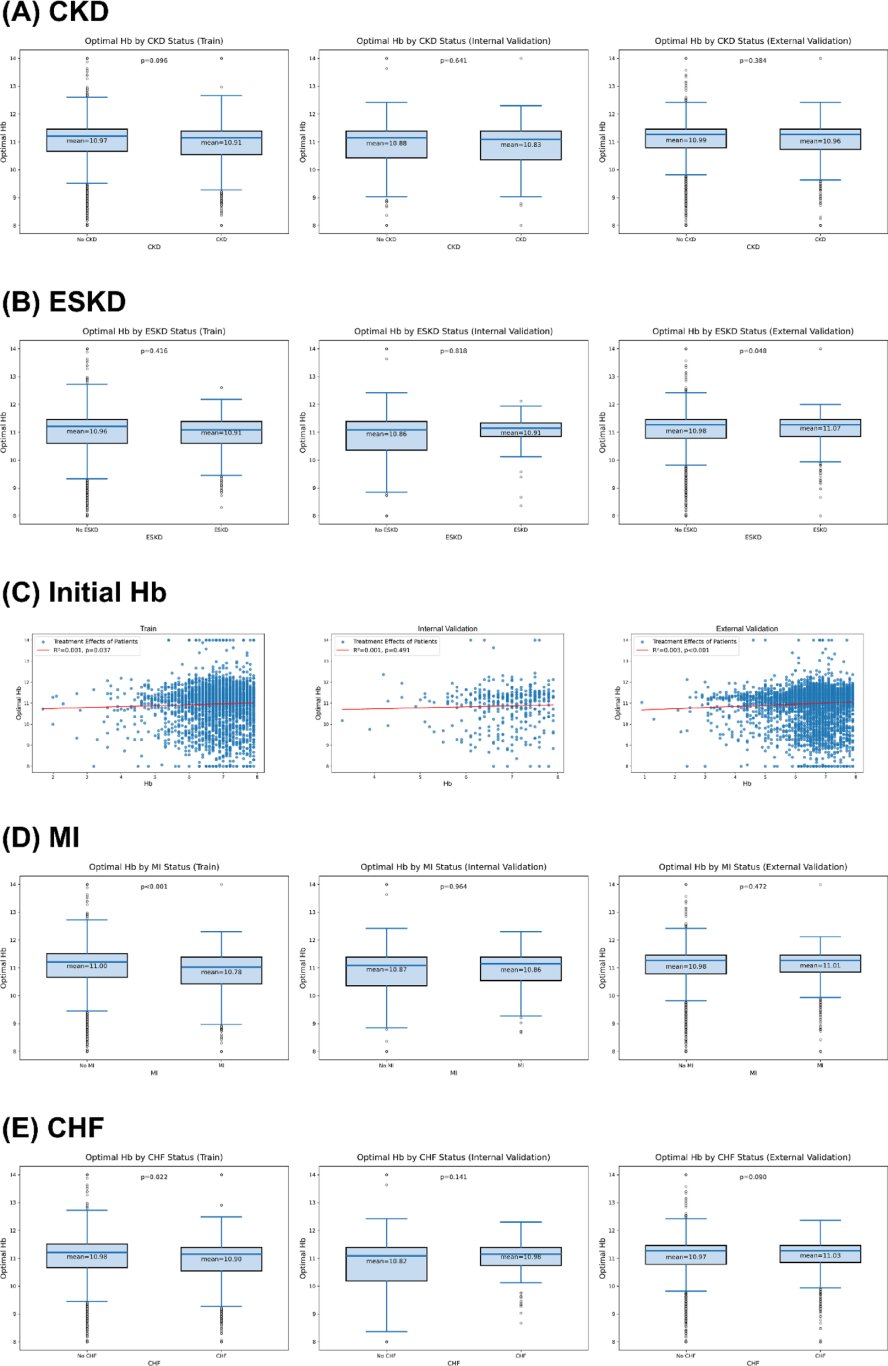

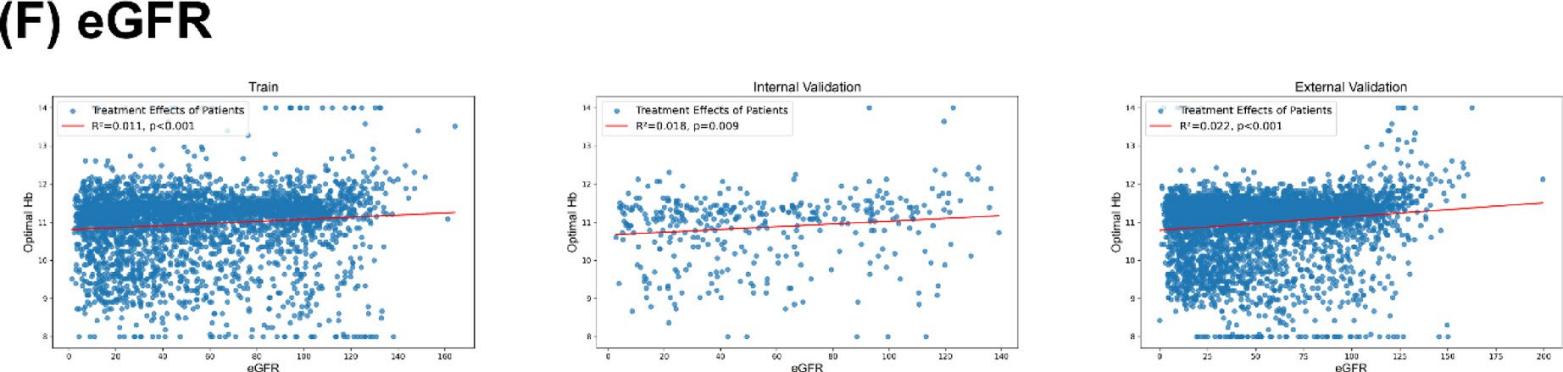
Subgroup comparisons and continuous relationships with the individualized optimal hemoglobin targets. Hb, hemoglobin; CKD, chronic kidney disease; ESKD, end-stage kidney disease; MI, myocardial infarction; CHF, congestive heart failure; eGFR, estimated glomerular filtration rate; (**A**), Chronic kidney disease. (**B**), End-stage kidney disease. (**C**), Initial hemoglobin. (**D**), Myocardial infarction. (**E**), Congestive heart failure. (**F**), estimated glomerular filtration rate.

Box-plot comparisons of mean optimal hemoglobin between admissions with and without CKD, ESKD, MI, or CHF confirmed uniformity, with mean targets ranging from 10.7 to 11.0 g/dL, and no comorbidity subgroup reaching statistical significance in any data (Fig. 3). Linear regression analyses (Fig. 3c) confirmed that admissions with lower initial hemoglobin had correspondingly lower optimal hemoglobin (training: R² = 0.001, *p* = 0.037; external validation: R² = 0.003, *p* < 0.001). In addition, lower eGFR was associated with lower optimal hemoglobin across all datasets (Fig. 3f), achieving significance in the training (R² = 0.011, *p* < 0.001), test (R² = 0.018, *p* = 0.009), and external validation (R² = 0.022, *p* < 0.001) data.

### Clinical predictors of predicted optimal hemoglobin targets below 10.0 g/dl

In the training data, an estimated optimal achieved post-transfusion hemoglobin level below 10.0 g/dL was associated with lower SBP, higher body temperature, lower average DBP, elevated WBC and platelet counts, lower pH, increased lactate levels, and higher doses of both norepinephrine and vasopressin (Supplementary Figure S5). In the test data, similar associations were observed for elevated WBC count, increased lactate concentration, and higher vasopressin requirements. In the external validation data, estimated optimal achieved post-transfusion hemoglobin level below 10.0 g/dL correlated with lower SBP, higher body temperature, lower average DBP, elevated WBC and platelet counts, lower pH, increased lactate, and greater requirements for norepinephrine and vasopressin.

## Discussion

In this study, we utilized a double machine learning-based causal inference framework to determine the optimal achieved post-transfusion hemoglobin level associated with the lowest in-hospital mortality among ICU patients. Our analysis revealed a consistent U-shaped relationship between post-transfusion hemoglobin and mortality across training, test, and external validation data, with an estimated optimal achieved post-transfusion hemoglobin level consistently around 11.0 g/dL. Moreover, the outcome prediction submodel demonstrated strong discrimination and calibration in both internal and external cohorts. Subgroup analyses demonstrated notable consistency, with estimated optimal levels remaining stable across patients with or without CKD, ESKD, MI, CHF, hypertension, and diabetes. Additionally, lower initial hemoglobin and eGFR at ICU admission were associated with lower individualized estimated optimal levels. Clinical characteristics commonly associated with systemic inflammation and hemodynamic instability—such as lower blood pressure, higher temperature, elevated WBC counts, increased lactate levels, and greater vasopressor requirements—were associated with an estimated optimal achieved post-transfusion hemoglobin level below 10.0 g/dL.

The integration of a double machine learning framework with causal forests enabled us to flexibly adjust for a rich set of confounders while estimating patient-specific treatment effects, uncovering the U-shaped relationship between post-transfusion hemoglobin and mortality^15^. Orthogonalizing the treatment and outcome models reduced bias from nuisance parameter estimation^25^, and the tree-based structure of causal forests captured nonlinear, heterogeneous responses across diverse ICU patients^15^. This combination maximized our ability to identify individualized optimal hemoglobin targets from observational data, adapting to complex patient characteristics. Nonetheless, these findings remain hypothesis-generating, and definitive confirmation through prospective randomized controlled trials is warranted.

Previous transfusion-related studies have largely focused on comparing liberal transfusion strategies (initiating transfusion at hemoglobin thresholds of approximately 9.0–10.0 g/dL) with restrictive strategies (thresholds of approximately 7.0–8.0 g/dL), often concluding no significant differences in outcomes between these approaches^3,5,8^. However, these studies generally did not distinguish clearly between transfusion initiation thresholds and optimal achieved post-transfusion hemoglobin levels. Our study uniquely addresses this gap by starting transfusions according to restrictive criteria (hemoglobin ≤ 8.0 g/dL) but subsequently exploring a broader range of hemoglobin levels post-transfusion. Our findings suggest that after initiating transfusion at restrictive thresholds, achieving a more liberal achieved post-transfusion hemoglobin level around 11.0 g/dL may be associated with improved survival outcomes. This nuanced approach represents a novel contribution to transfusion management literature, highlighting the importance of differentiating between initiation thresholds and therapeutic goals in critically ill anemic patients. One potential explanation for our findings is that separating the transfusion trigger from the maintenance goal allows clinicians to balance safety and efficacy more effectively. By adhering to a restrictive initiation threshold (hemoglobin ≤ 8.0 g/dL), we minimized unnecessary exposure to transfusion-related risks, while subsequently targeting a moderately higher hemoglobin—around 11.0 g/dL—to better satisfy the elevated metabolic demands of critically ill patients and enhance tissue oxygenation^26,27^.

It is important to contextualize our findings against major randomized controlled trials. The Transfusion Requirements in Critical Care (TRICC) trial demonstrated noninferiority of a restrictive strategy (hemoglobin trigger < 7.0 g/dL) compared with a liberal strategy (< 10.0 g/dL), with achieved post-transfusion hemoglobin levels in the restrictive group typically around 8.5 g/dL^3^. Similarly, the Transfusion Requirements in Septic Shock (TRISS) trial found no significant difference in 90-day mortality between restrictive (trigger ≤ 7.0 g/dL) and liberal (trigger ≤ 9.0 g/dL) thresholds^28^. In both trials, the restrictive groups achieved relatively low post-transfusion hemoglobin levels (approximately 7.5–8.5 g/dL), yet outcomes were noninferior. Our observational finding that an achieved hemoglobin around 11.0 g/dL is associated with the lowest predicted mortality may appear to conflict with these results. However, several factors may account for this discrepancy. Our study population was restricted to patients with pre-transfusion hemoglobin ≤ 8.0 g/dL who received a single early transfusion episode, representing a different clinical scenario from the repeated transfusion protocols in these trials. Moreover, our analysis examines the achieved post-transfusion hemoglobin as a continuous exposure rather than a binary threshold comparison, which may capture dose–response relationships not detectable in conventional two-arm trial designs. Nonetheless, these discrepancies underscore the hypothesis-generating nature of our findings and the necessity of prospective validation.

Subgroup analyses indicated that estimated optimal achieved post-transfusion hemoglobin levels remained consistently around 11.0 g/dL across major comorbidity subgroups. The observed consistency suggests the potential generalizability of our findings within ICU settings, including patients with CKD, ESKD, MI, CHF, hypertension, and diabetes. Lower initial hemoglobin and reduced eGFR at ICU admission were associated with lower individualized estimated optimal levels. This pattern may reflect a clinical strategy to limit additional transfusion volume—and thereby mitigate the elevated risk of volume overload and other transfusion-related complications—in patients presenting with more severe anemia or impaired kidney function^29,30^.

An important consideration is the potential for post-treatment bias inherent in our study design. Because the achieved post-transfusion hemoglobin is measured after transfusion, it may reflect not only the intended clinical goal but also evolving illness severity and treatment response. Patients with active, brisk bleeding—particularly those in hemorrhagic shock—may fail to achieve higher hemoglobin levels despite receiving transfusions, owing to ongoing blood loss. These patients typically present with higher vasopressor requirements, lower blood pressure, metabolic acidosis, and elevated lactate—features that are independently associated with worse outcomes. Conversely, once hemorrhage is controlled, these patients may attain relatively high post-transfusion hemoglobin levels and tend to have more favorable survival prospects than patients with sepsis-driven anemia. This differential prognosis across shock etiologies may therefore confound the observed association between higher achieved hemoglobin and lower mortality. Furthermore, in septic shock, transfusion is typically guided by restrictive thresholds, with achieved hemoglobin remaining in the 7.0–9.0 g/dL range, whereas in hemorrhagic shock, resuscitation guided by perfusion markers may result in higher achieved levels after bleeding control. We attempted to reduce post-treatment bias by restricting the cohort, defining the exposure according to its temporal relationship to transfusion, and adjusting for early severity-related covariates; however, because some first-24-hour summaries may have included measurements obtained after transfusion, residual post-treatment bias cannot be fully excluded. Accordingly, our findings should be interpreted as observational, hypothesis-generating evidence rather than as estimates of the causal effect of assigning a fixed post-transfusion hemoglobin level.

Our analysis found that lower estimated optimal achieved post-transfusion hemoglobin levels (< 10.0 g/dL) tended to occur in patients with signs of severe inflammation and sepsis—hypotension, fever, leukocytosis, elevated lactate, acidosis, and high vasopressor requirements. In such cases, sepsis-induced capillary leak and interstitial edema may blunt the benefit of higher intravascular hemoglobin by impairing microvascular oxygen diffusion^31,32^. Additionally, transfusion‐related immunomodulation—via leukocyte cytokine release and complement activation—can further amplify systemic inflammation^33,34^. Taken together, these mechanisms suggest that more modest achieved post-transfusion hemoglobin levels may be preferable in sepsis-like states to avoid exacerbating volume overload and inflammatory injury.

Several limitations should be acknowledged. First, as discussed above, the observational design and post-transfusion measurement of the exposure introduce the possibility of post-treatment bias and confounding by bleeding severity and shock etiology, and our findings should be interpreted as hypothesis-generating rather than causal. Second, our exclusion of patients who received additional transfusions after 48 h may introduce selection bias by preferentially removing more unstable or persistently bleeding patients from the analysis. Third, although we conducted external validation using the eICU database, residual confounding may persist due to unmeasured variables such as the number of transfused units, bleeding source, and shock etiology. The eICU dataset also contained substantial missing data, potentially limiting validation robustness, and we acknowledge that validation on additional independent databases (e.g., AmsterdamUMCdb, HiRID, SICdb) would further strengthen generalizability. Fourth, the 90:10 train–test split may raise concerns about overfitting, although external validation on the eICU cohort provides a degree of reassurance. Fifth, key causal assumptions—unconfoundedness, overlap, and the stable unit treatment value assumption (SUTVA)—may not be fully satisfied in observational ICU data, and violations could bias our treatment effect estimates. Lastly, our analyses depended on retrospective electronic medical records, which can contain inaccuracies or omissions, and the model’s clinical applicability should be confirmed prospectively.

In conclusion, our causal-inference analysis of two large ICU data suggests that, after initiating transfusion at a restrictive threshold (hemoglobin ≤ 8.0 g/dL), an achieved post-transfusion hemoglobin of approximately 11.0 g/dL was associated with the lowest predicted in-hospital mortality in our models. While this finding suggests the potential value of decoupling the transfusion trigger from the post-transfusion hemoglobin goal, the observational design, potential for residual confounding (including bleeding severity and shock etiology), and limitations inherent to retrospective database analyses preclude definitive causal conclusions. These hypothesis-generating findings require confirmation in prospective, randomized settings to establish causality, assess safety, and determine generalizability across different ICU subpopulations.

## Electronic Supplementary Material

Below is the link to the electronic supplementary material.

Supplementary Material 1

## Data Availability

The datasets analyzed in the present study are publicly available and can be accessed through the eICU Collaborative Research Database and MIMIC-IV Database from PhysioNet.- eICU Collaborative Research Database: https://physionet.org/content/eicu-crd/2.0/- MIMIC-IV Database: https://physionet.org/content/mimiciv/3.1/.
