# Supplementary material for: Impact of post-transfusion hemoglobin levels on survival in critically Ill patients: a machine learning–based causal inference analysis
Source: Sci Rep. 2026 Apr 25;16:19225. doi: 10.1038/s41598-026-50363-y (PMC13284187; doi:10.1038/s41598-026-50363-y)

**Optimal Post-Transfusion Hemoglobin Targets in Critically Ill Patients: A Machine Learning–Based Causal Inference Analysis**

Min Woo Kang, Soojeong Yun, Seung Min Song, Ji Eun Kim, Hyo Jin Kim, Eun Jung Cho, Gang Jee Ko, Young Joo Kwon, and Shin Young Ahn

Supplementary Table S1-5

Supplementary Figure S1-5

Supplementary Table S1. Missing values in Medical Information Mart for Intensive Care-IV

| **Variable** | **Missing proportion (%)** |
| --- | --- |
| **Protein** | 96.66 |
| **CRP** | 96.55 |
| **Height** | 47.01 |
| **Albumin** | 46.90 |
| **ALT** | 29.54 |
| **AST** | 28.49 |
| **Total bilirubin** | 27.82 |
| **pH** | 18.52 |
| **Lactate** | 17.17 |
| **Weight** | 11.48 |
| **aPTT** | 4.77 |
| **PT INR** | 4.39 |
| **Temperature** | 1.35 |
| **Calcium** | 0.38 |
| **SpO_2_** | 0.27 |
| **SBP** | 0.11 |
| **DBP** | 0.05 |
| **WBC** | 0.05 |
| **Platelet** | 0.05 |
| **Bicarbonate** | 0.03 |
| **eGFR** | 0.03 |
| **Heart rate** | 0.03 |
| **Anion gap** | 0.03 |
| **Sodium** | 0.03 |
| **Potassium** | 0.03 |
| **Creatinine** | 0.03 |

Abbreviation: CRP, C-reactive protein; ALT, alanine aminotransferase; AST, aspartate aminotransferase; aPTT, activated partial thromboplastin time; PT INR, prothrombin time international normalized ratio; SpO₂, saturation of peripheral oxygen; SBP, systolic blood pressure; DBP diastolic blood pressure; WBC, white blood cell; eGFR, estimated glomerular filtration rate.

Supplementary Table S2. Missing values in eICU

| **Variable** | **Missing proportion (%)** |
| --- | --- |
| **CRP** | 97.16 |
| **Temperature** | 87.43 |
| **SBP** | 64.19 |
| **DBP** | 64.02 |
| **Average DBP** | 63.47 |
| **Average SBP** | 63.42 |
| **Lactate** | 48.12 |
| **pH** | 43.08 |
| **aPTT** | 35.03 |
| **Total bilirubin** | 28.35 |
| **ALT** | 25.9 |
| **Protein** | 25.83 |
| **AST** | 24.84 |
| **Albumin** | 21.85 |
| **PT INR** | 17.8 |
| **Antion gap** | 4.11 |
| **Bicarbonate** | 3.71 |
| **Weight** | 3.24 |
| **eGFR** | 3.22 |
| **Baseline eGFR** | 3.12 |
| **Age** | 2.90 |
| **SpO_2_** | 2.42 |
| **Calcium** | 1.48 |
| **Heart rate** | 0.93 |
| **Platelet** | 0.91 |
| **Height** | 0.85 |
| **WBC** | 0.34 |
| **Creatinine** | 0.32 |
| **BUN** | 0.32 |
| **Chloride** | 0.21 |
| **Baseline creatinine** | 0.21 |
| **Sodium** | 0.15 |
| **Potassium** | 0.11 |

Abbreviation: CRP, C-reactive protein; SBP, systolic blood pressure; DBP diastolic blood pressure; aPTT, activated partial thromboplastin time; ALT, alanine aminotransferase; AST, aspartate aminotransferase; PT INR, prothrombin time international normalized ratio; eGFR, estimated glomerular filtration rate; SpO₂, saturation of peripheral oxygen; WBC, white blood cell; BUN, blood urea nitrogen.

Supplementary Table S3. Complete list of baseline covariates, definitions, and summary methods

| Variable | Definition | Unit | Summary |
| --- | --- | --- | --- |
| *Demographic characteristics* | | | |
| Age | Age at ICU admission | Years | — |
| Sex | Biological sex | Male/Female | — |
| Weight | Body weight at admission | kg | — |
| Height | Body height at admission | cm | — |
| *Vital signs (first 24 hours)* | | | |
| Heart rate | Maximum heart rate | beats/min | Maximum |
| SBP | Minimum systolic blood pressure | mmHg | Minimum |
| DBP | Minimum diastolic blood pressure | mmHg | Minimum |
| Average SBP | 24-hour average systolic blood pressure | mmHg | — |
| Average DBP | 24-hour average diastolic blood pressure | mmHg | — |
| Temperature | Maximum body temperature | °C | Maximum |
| SpO₂ | Minimum peripheral oxygen saturation | % | Minimum |
| *Respiratory support* | | | |
| Mechanical ventilation | Use of invasive mechanical ventilation | Yes/No | — |
| FiO₂ | Maximum fraction of inspired oxygen | % | Maximum |
| *Laboratory measurements (first 24 hours)* | | | |
| Initial hemoglobin | Nadir pre-transfusion hemoglobin within 24 h of ICU admission | g/dL | Minimum |
| Creatinine | Maximum serum creatinine | mg/dL | Maximum |
| eGFR | Minimum estimated glomerular filtration rate (CKD-EPI equation) | mL/min/1.73 m² | Minimum |
| Baseline creatinine | Lowest creatinine within 6 months pre-ICU (or initial ICU value if unavailable) | mg/dL | — |
| Baseline eGFR | eGFR derived from baseline creatinine | mL/min/1.73 m² | — |
| AST | Maximum aspartate aminotransferase | U/L | Maximum |
| ALT | Maximum alanine aminotransferase | U/L | Maximum |
| Total bilirubin | Maximum total bilirubin | mg/dL | Maximum |
| WBC | Maximum white blood cell count | 10³/µL | Maximum |
| Platelet | Minimum platelet count | 10³/µL | Minimum |
| Calcium | Maximum serum calcium | mg/dL | Maximum |
| pH | Minimum arterial pH | — | Minimum |
| Bicarbonate | Minimum serum bicarbonate | mmol/L | Minimum |
| Anion gap | Maximum anion gap | mmol/L | Maximum |
| BUN | Maximum blood urea nitrogen | mg/dL | Maximum |
| Sodium | Minimum serum sodium | mmol/L | Minimum |
| Potassium | Maximum serum potassium | mmol/L | Maximum |
| Chloride | Minimum serum chloride | mmol/L | Minimum |
| PT–INR | Maximum prothrombin time–international normalized ratio | — | Maximum |
| aPTT | Maximum activated partial thromboplastin time | sec | Maximum |
| Lactate | Maximum serum lactate | mmol/L | Maximum |
| *Vasoactive and inotropic agents (first 24 hours)* | | | |
| Norepinephrine | Maximum infusion rate | mcg/kg/min | Maximum |
| Dopamine | Maximum infusion rate | mcg/kg/min | Maximum |
| Epinephrine | Maximum infusion rate | mcg/kg/min | Maximum |
| Vasopressin | Maximum infusion rate | units/hr | Maximum |
| Dobutamine | Maximum infusion rate | mcg/kg/min | Maximum |
| *Comorbidities (ICD-9/10 codes)* | | | |
| CKD | Chronic kidney disease | Yes/No | — |
| ESKD | End-stage kidney disease | Yes/No | — |
| MI | Myocardial infarction | Yes/No | — |
| CHF | Congestive heart failure | Yes/No | — |
| PVD | Peripheral vascular disease | Yes/No | — |
| CVD | Cerebrovascular disease | Yes/No | — |
| Hypertension | Hypertension | Yes/No | — |
| Diabetes | Diabetes mellitus | Yes/No | — |
| Chronic liver disease | Chronic liver disease | Yes/No | — |
| *Other* | | | |
| Non-cardiac surgery | Receipt of non-cardiac surgery within 3 days of ICU admission | Yes/No | — |
| RRT | Renal replacement therapy within 48 hours | Yes/No | — |

Abbreviations: ICU, intensive care unit; SBP, systolic blood pressure; DBP, diastolic blood pressure; SpO₂, peripheral oxygen saturation; FiO₂, fraction of inspired oxygen; eGFR, estimated glomerular filtration rate; CKD-EPI, Chronic Kidney Disease Epidemiology Collaboration; AST, aspartate aminotransferase; ALT, alanine aminotransferase; WBC, white blood cell; BUN, blood urea nitrogen; PT–INR, prothrombin time–international normalized ratio; aPTT, activated partial thromboplastin time; CKD, chronic kidney disease; ESKD, end-stage kidney disease; MI, myocardial infarction; CHF, congestive heart failure; PVD, peripheral vascular disease; CVD, cerebrovascular disease; RRT, renal replacement therapy; ICD, International Classification of Diseases.

Supplementary Table S4. Baseline characteristics of train and test data

| **Variable** | **Train**  **(N = 3339)** | **Test**  **(N = 371)** | **p-value** |
| --- | --- | --- | --- |
| **Age (years)** | 63.76 ± 15.43 | 62.97 ± 16.07 | 0.355 |
| **Weight (kg)** | 77.60 ± 19.63 | 77.42 ± 22.42 | 0.868 |
| **Heart rate (beats/min)** | 113.94 ± 21.63 | 115.33 ± 22.08 | 0.242 |
| **SBP (mmHg)** | 82.69 ± 15.44 | 83.16 ± 15.36 | 0.58 |
| **DBP (mmHg)** | 40.42 ± 9.94 | 40.08 ± 9.95 | 0.531 |
| **Temperature (°C)** | 37.57 ± 0.75 | 37.63 ± 0.79 | 0.161 |
| **SpO₂ (%)** | 89.70 ± 7.42 | 89.61 ± 6.91 | 0.824 |
| **Average SBP (mmHg)** | 114.09 ± 15.33 | 114.67 ± 15.04 | 0.488 |
| **Average DBP (mmHg)** | 59.55 ± 9.85 | 59.60 ± 10.36 | 0.923 |
| **FiO₂ (%)** | 53.97 ± 30.90 | 57.53 ± 31.04 | 0.035 |
| **WBC (10³/µL)** | 15.64 ± 16.16 | 16.13 ± 10.76 | 0.569 |
| **Initial hemoglobin (g/dL)** | 6.80 ± 0.80 | 6.85 ± 0.74 | 0.297 |
| **Platele (10³/µL)** | 165.34 ± 125.66 | 173.77 ± 126.10 | 0.220 |
| **ALT (U/L)** | 138.19 ± 460.47 | 149.62 ± 670.45 | 0.667 |
| **AST (U/L)** | 249.27 ± 894.70 | 320.30 ± 1914.33 | 0.213 |
| **Total bilirubin (mg/dL)** | 2.92 ± 6.05 | 2.98 ± 5.67 | 0.871 |
| **Calcium (mg/dL)** | 8.57 ± 1.11 | 8.49 ± 1.12 | 0.219 |
| **pH** | 7.32 ± 0.11 | 7.31 ± 0.10 | 0.549 |
| **Bicarbonate (mmol/L)** | 20.22 ± 5.10 | 20.45 ± 4.86 | 0.390 |
| **Anion gap (mmol/L)** | 17.09 ± 5.35 | 16.96 ± 4.86 | 0.649 |
| **BUN (mg/dL)** | 41.01 ± 30.23 | 40.36 ± 29.51 | 0.694 |
| **Creatinine (mg/dL)** | 2.13 ± 2.18 | 2.09 ± 2.22 | 0.754 |
| **Sodium (mmol/L)** | 135.73 ± 5.89 | 135.58 ± 5.81 | 0.657 |
| **Potassium (mmol/L)** | 4.70 ± 0.84 | 4.70 ± 0.75 | 0.910 |
| **Chloride (mmol/L)** | 101.17 ± 7.18 | 101.08 ± 7.00 | 0.818 |
| **PT INR** | 1.87 ± 1.15 | 1.85 ± 1.21 | 0.84 |
| **aPTT (sec)** | 51.03 ± 33.83 | 50.96 ± 35.00 | 0.968 |
| **Lactate (mmol/L)** | 3.06 ± 2.71 | 3.26 ± 2.77 | 0.188 |
| **Baseline creatinine (mg/dL)** | 1.71 ± 1.97 | 1.65 ± 1.83 | 0.535 |
| **eGFR (mL/min/1.73 m²)** | 55.20 ± 35.26 | 55.21 ± 35.13 | 0.997 |
| **Baseline eGFR (mL/min/1.73 m²)** | 67.22 ± 37.73 | 65.72 ± 36.44 | 0.464 |
| **Norepinephrine (mcg/kg/min)** | 0.05 ± 0.15 | 0.04 ± 0.10 | 0.413 |
| **Dopamine (mcg/kg/min)** | 0.16 ± 1.29 | 0.38 ± 2.28 | 0.005 |
| **Epinephrine (mcg/kg/min)** | 0.00 ± 0.05 | 0.00 ± 0.05 | 0.732 |
| **Vasopressin (units/hr)** | 0.27 ± 0.84 | 0.29 ± 0.78 | 0.659 |
| **Dobutamine (mcg/kg/min)** | 0.05 ± 0.49 | 0.05 ± 0.42 | 0.869 |
| **Target hemoglobin (g/dL)** | 8.96 ± 1.26 | 9.14 ± 1.38 | 0.012 |
| **Male** | 1773 (53.1%) | 174 (46.9%) | >0.999 |
| **RRT within 48 hours** | 80 (2.4%) | 8 (2.2%) | >0.999 |
| **Mechanical ventilation** | 1829 (54.8%) | 229 (61.7%) | >0.999 |
| **Chronic kidney disease** | 774 (23.2%) | 88 (23.7%) | >0.999 |
| **End-stage kidney disease** | 277 (8.3%) | 32 (8.6%) | >0.999 |
| **Myocardial infarction** | 585 (17.5%) | 71 (19.1%) | >0.999 |
| **Congestive heart failure** | 1073 (32.1%) | 114 (30.7%) | >0.999 |
| **Peripheral vascular disease** | 512 (15.3%) | 67 (18.1%) | >0.999 |
| **Cerebrovascular disease** | 401 (12.0%) | 52 (14.0%) | >0.999 |
| **Chronic liver disease** | 883 (26.4%) | 104 (28.0%) | >0.999 |
| **Diabetes** | 1118 (33.5%) | 117 (31.5%) | >0.999 |
| **Hypertension** | 1445 (43.3%) | 164 (44.2%) | >0.999 |
| **Non-cardiac surgery** | 480 (14.4%) | 63 (17.0%) | >0.999 |
| **In-hospital mortality** | 602 (18.0%) | 67 (18.1%) | >0.999 |

Abbreviation: SBP, systolic blood pressure; DBP diastolic blood pressure; SpO₂, saturation of peripheral oxygen; FiO₂, fraction of inspired oxygen; WBC, white blood cell; ALT, alanine aminotransferase; AST, aspartate aminotransferase; BUN, blood urea nitrogen; PT INR, prothrombin time international normalized ratio; aPTT, activated partial thromboplastin time; eGFR, estimated glomerular filtration rate; RRT, renal replacement therapy.

Supplementary Table S5. Model performance metrics in test and external validation data

| **Metric** | **Test** | **External validation** |
| --- | --- | --- |
| **NN-PEHE** | 0.5134 | 0.4752 |
| **AUROC** | 0.7667 | 0.7655 |
| **Accuracy** | 0.8329 | 0.8813 |
| **Brier score** | 0.1249 | 0.0954 |

Abbreviation: NN-PEHE, nearest‐neighbor precision in estimating heterogeneous effects; AUROC, area under the receiver operating characteristic curve.

Supplementary Figure S1. Flow diagram of patient selection and study design


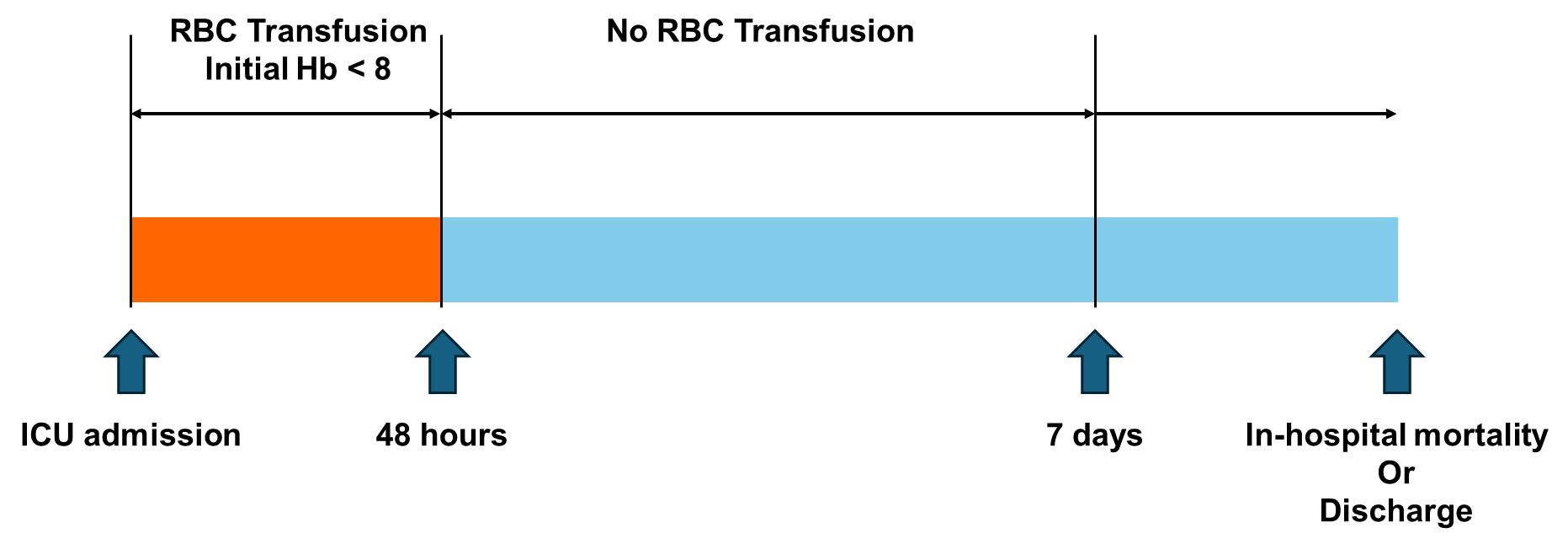


Supplementary Figure S2. Directed acyclic graph illustrating the assumed causal structure and potential for post-treatment bias. BP, blood pressure.


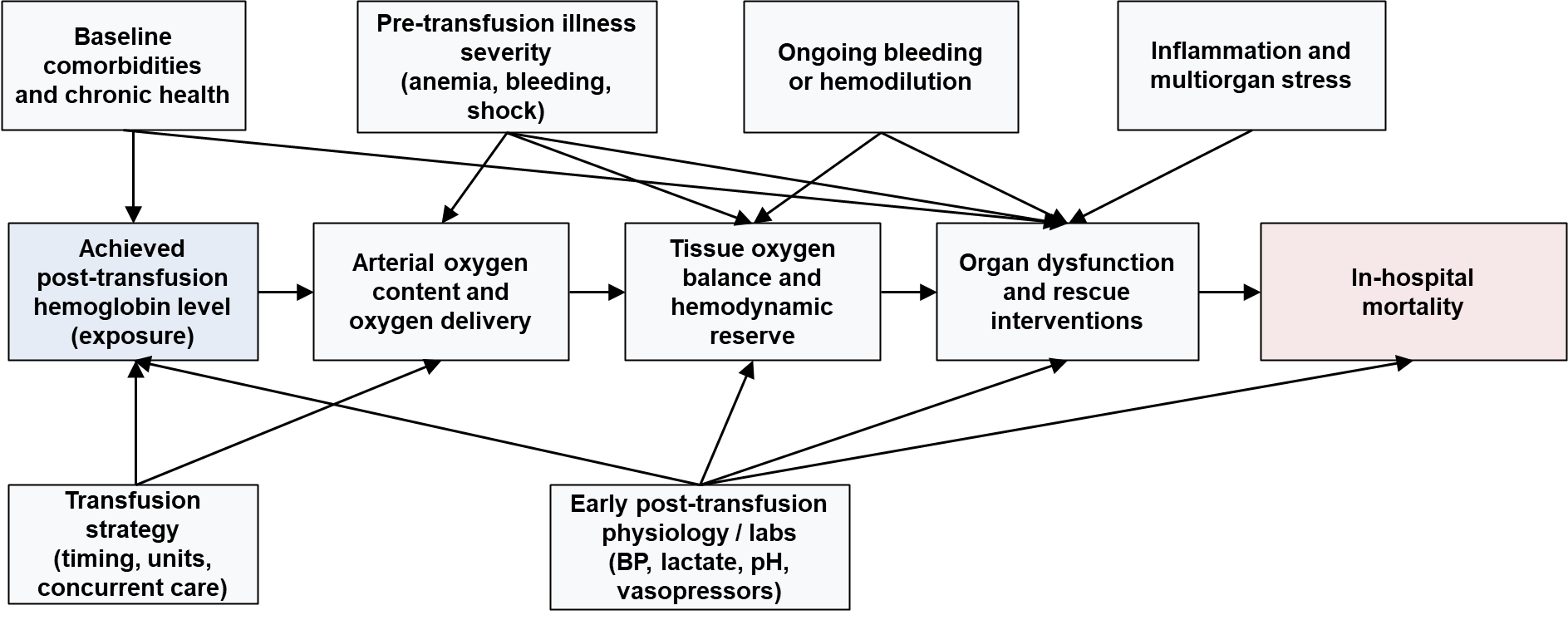


Supplementary Figure S3. Treatment-effect curves for post-transfusion hemoglobin re-centered on the individualized optimal hemoglobin level, with 95% confidence intervals. Hb, hemoglobin.

(A) Train data


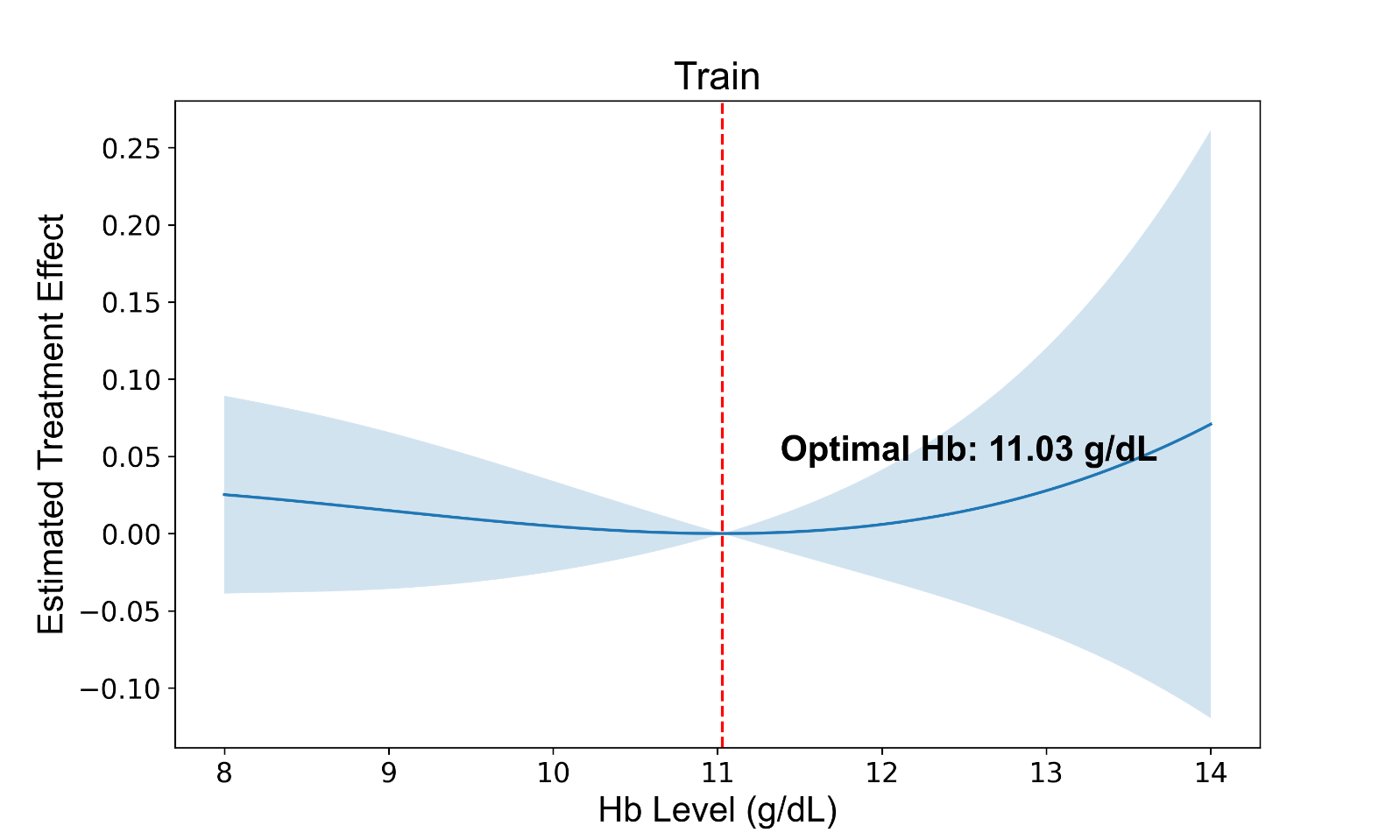


(B) Internal validation


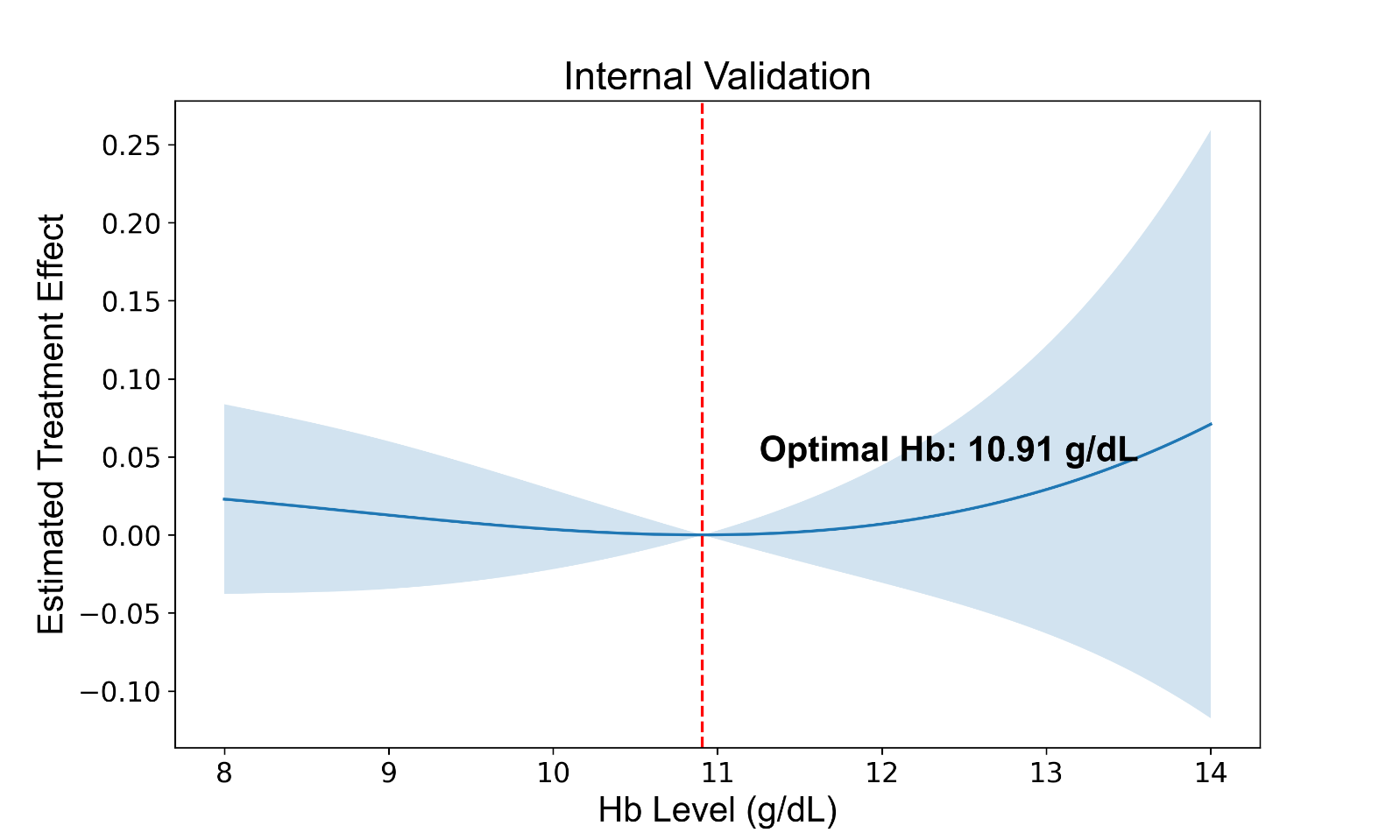


(C) External validation


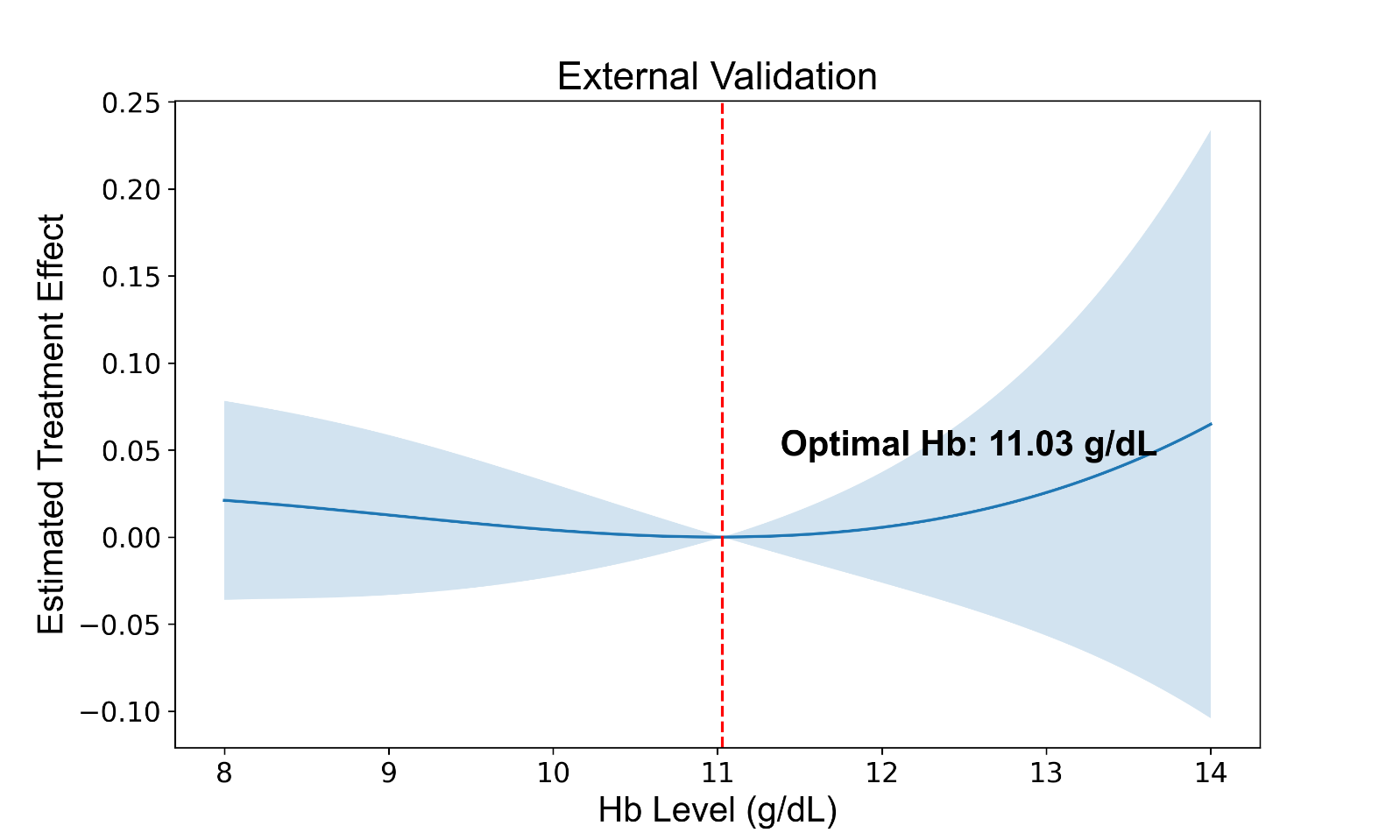


Supplementary Figure S4. Average treatment effect curves for post-transfusion hemoglobin targets stratified by comorbidity and initial hemoglobin strata. Hb, hemoglobin; CKD, chronic kidney disease; ESKD, end-stage kidney disease; MI, myocardial infarction; CHF, congestive heart failure; HTN, hypertension; (A), Chronic kidney disease. (B), End-stage kidney disease. (C), Initial hemoglobin. (D), Myocardial infarction. (E), Congestive heart failure. (F), Hypertension. (G), Diabetes.


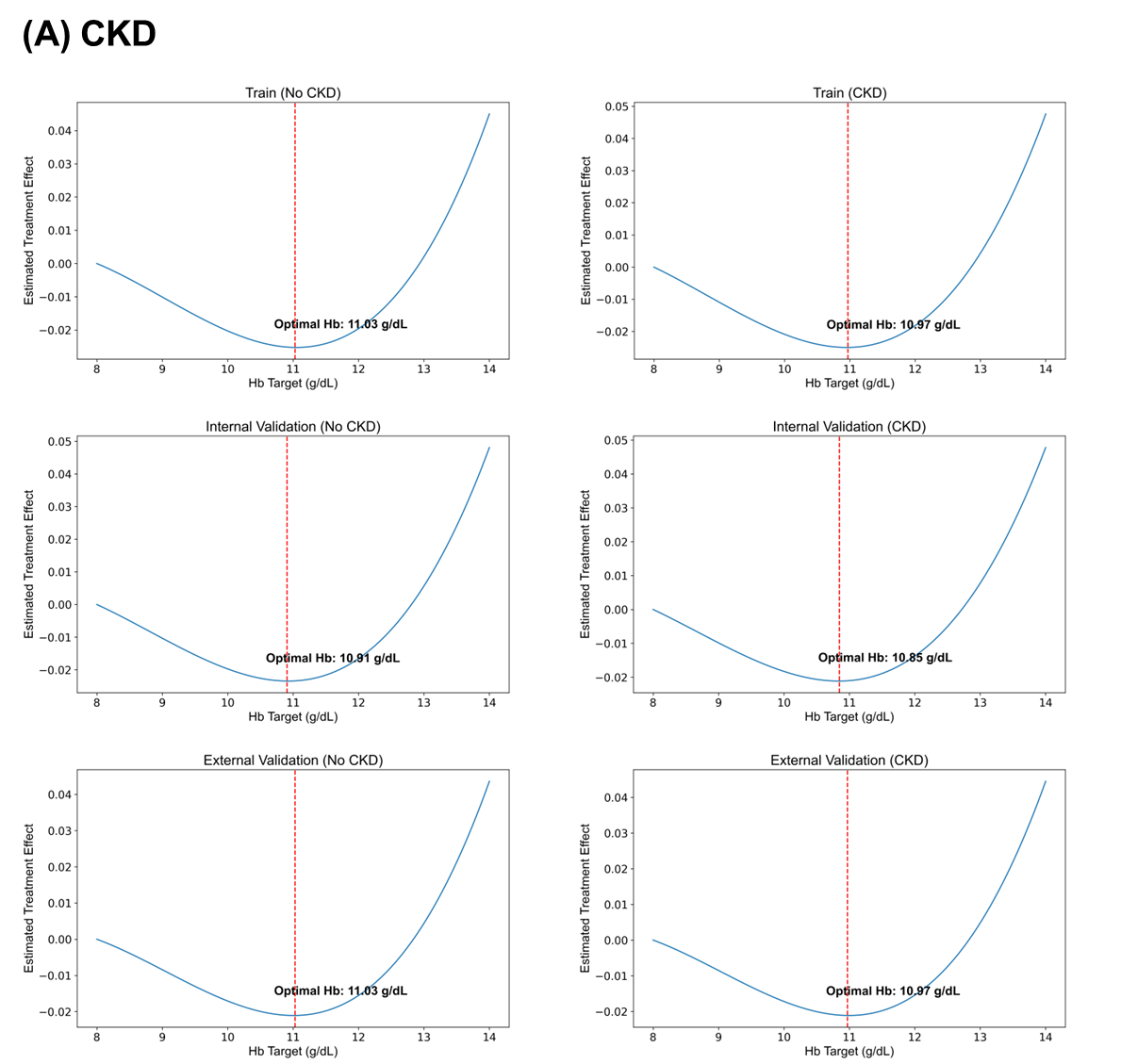


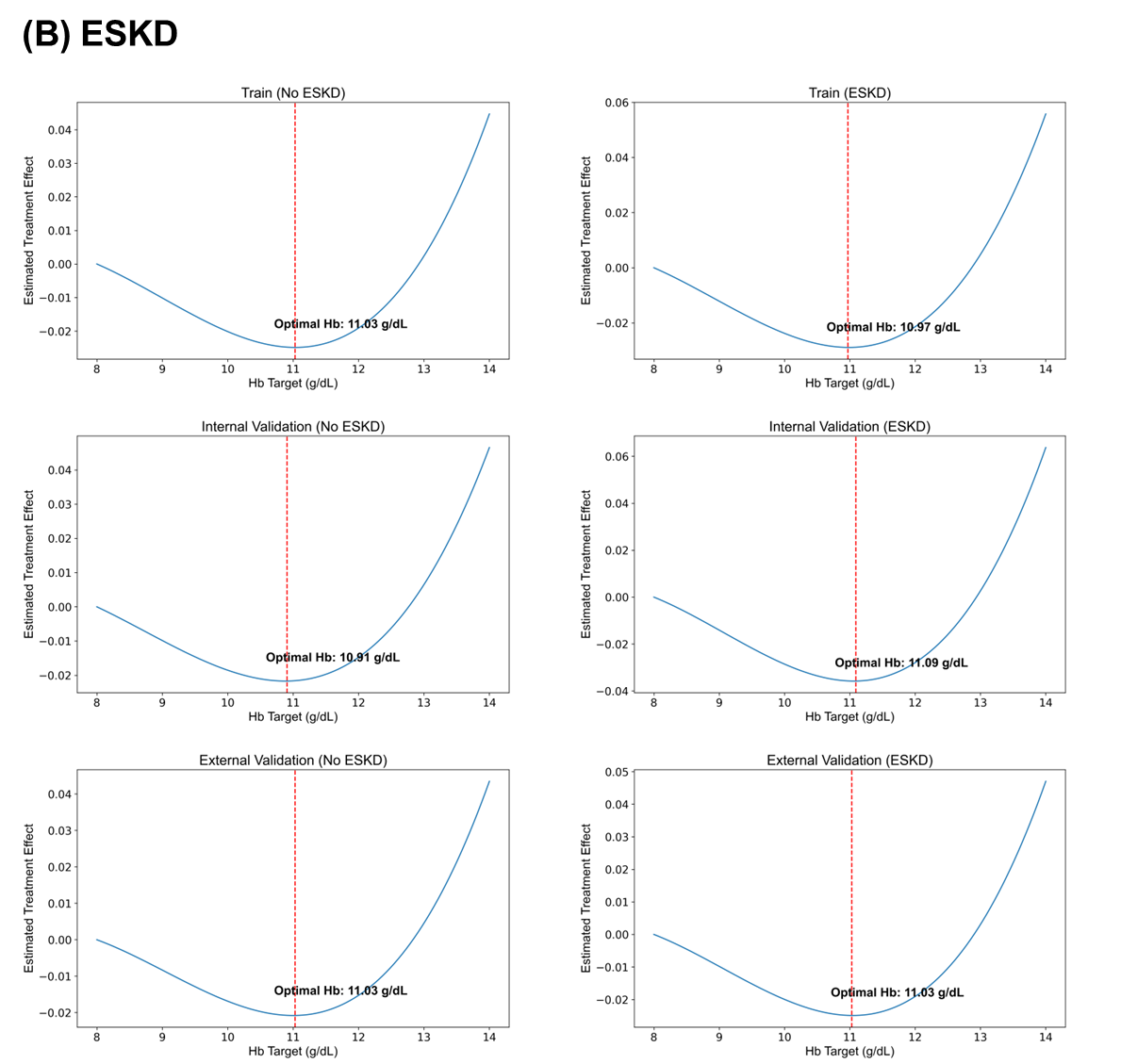


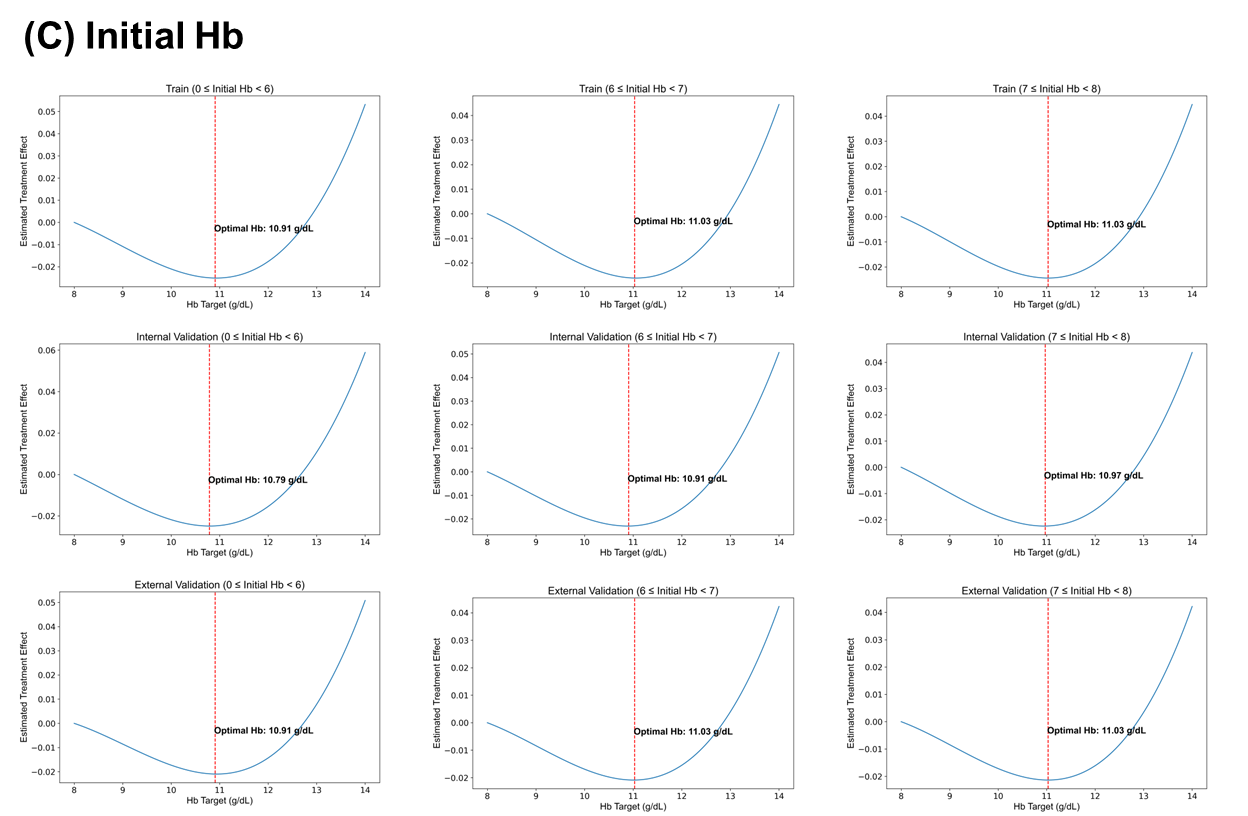

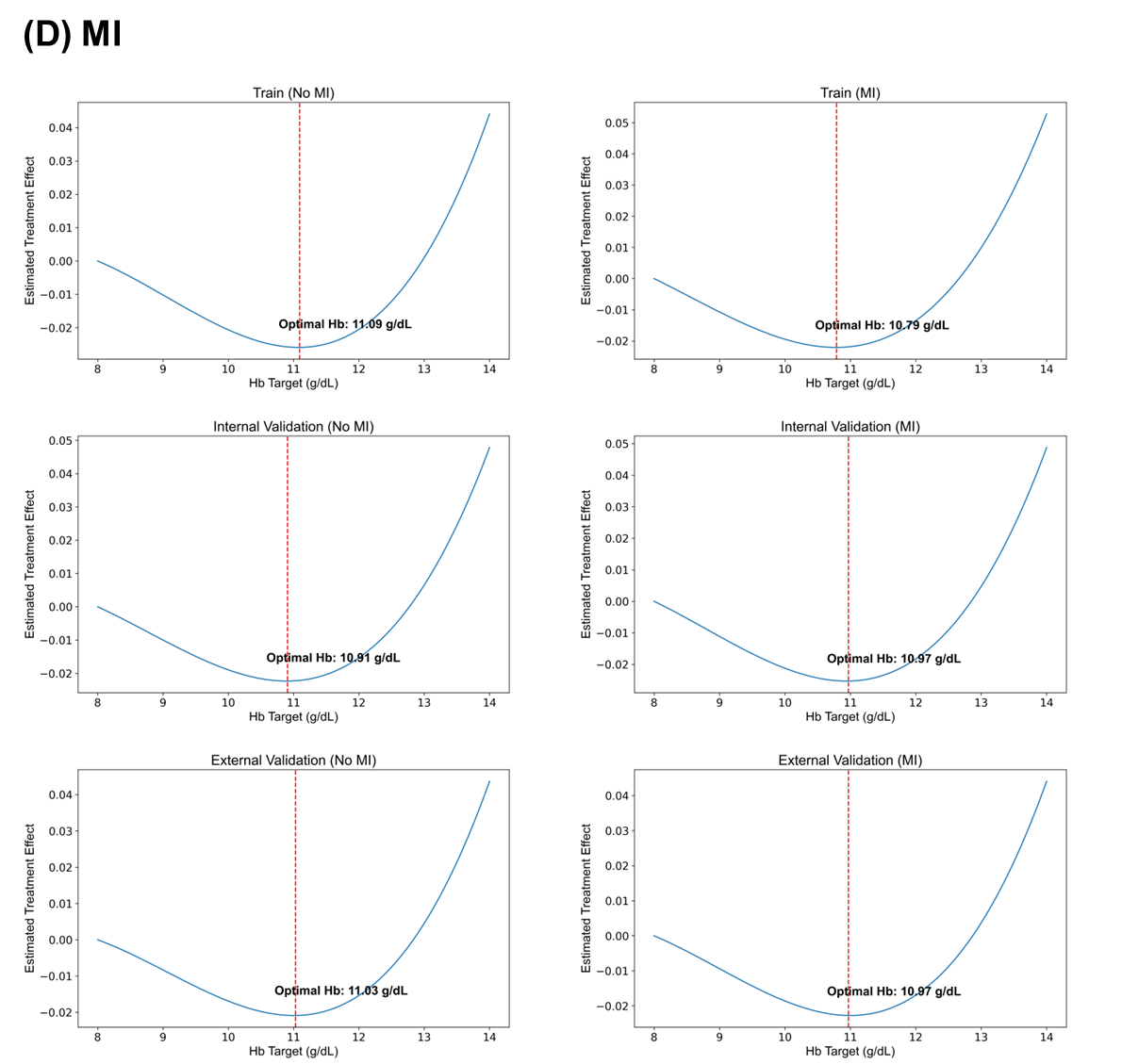

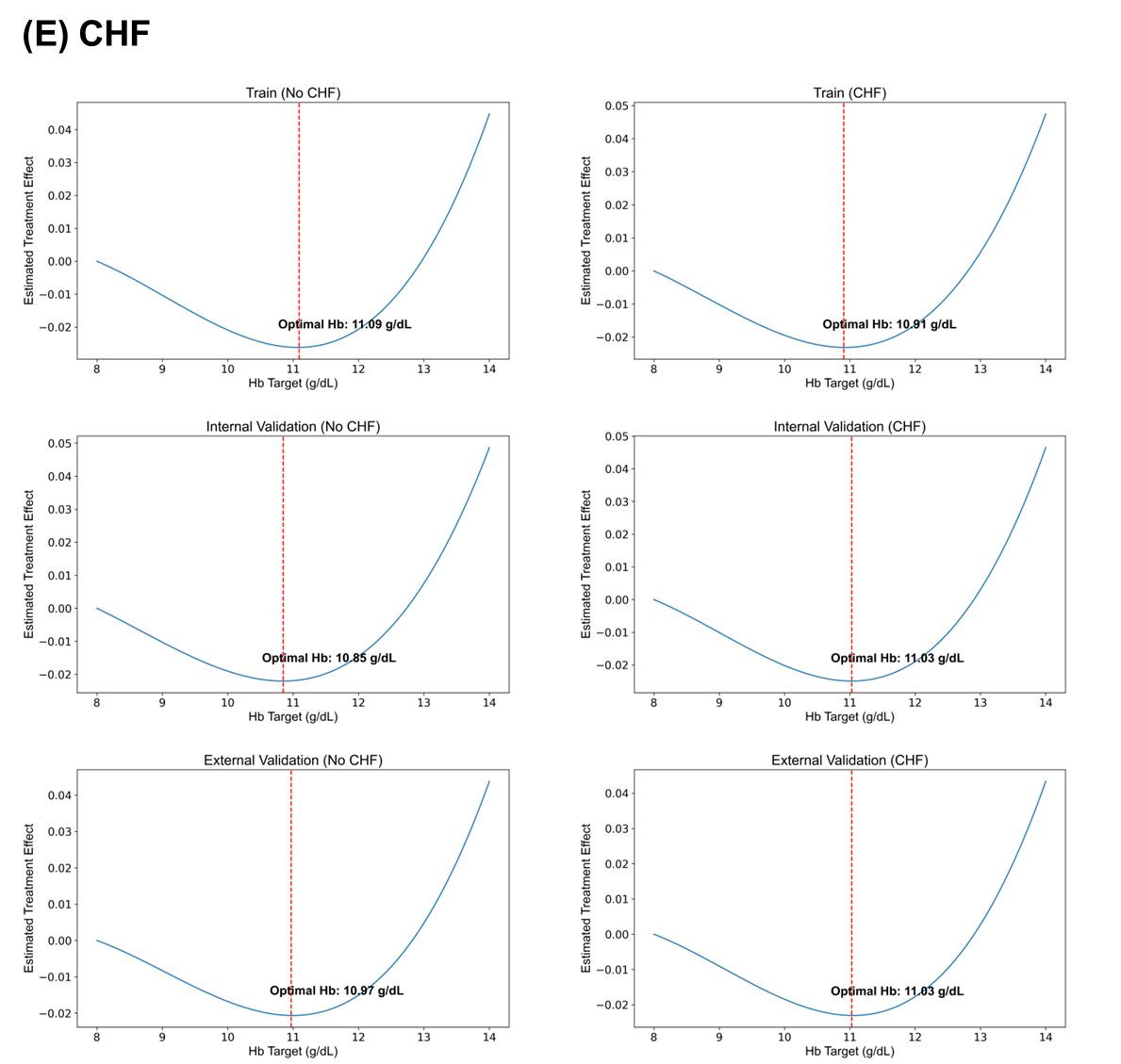

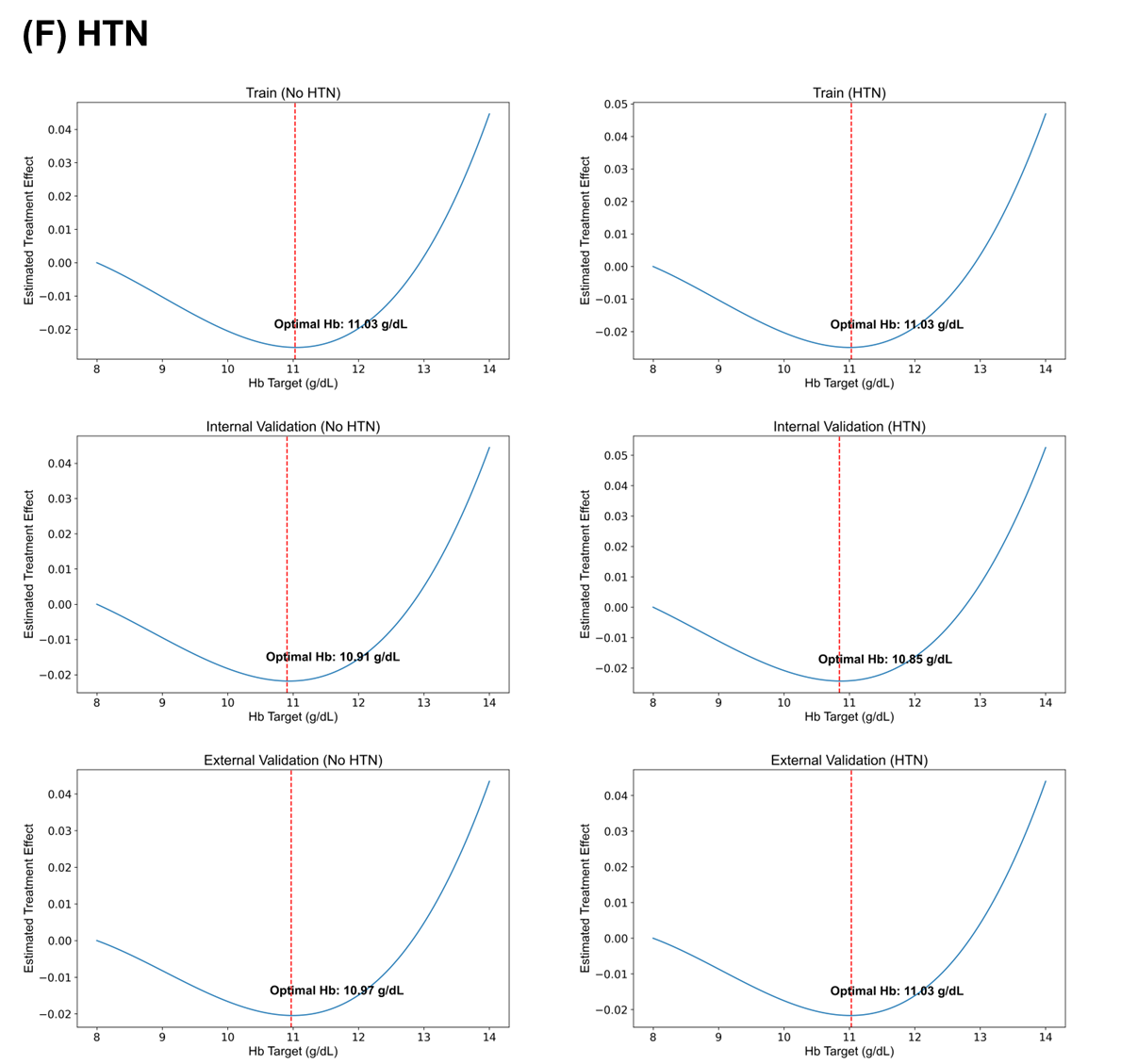

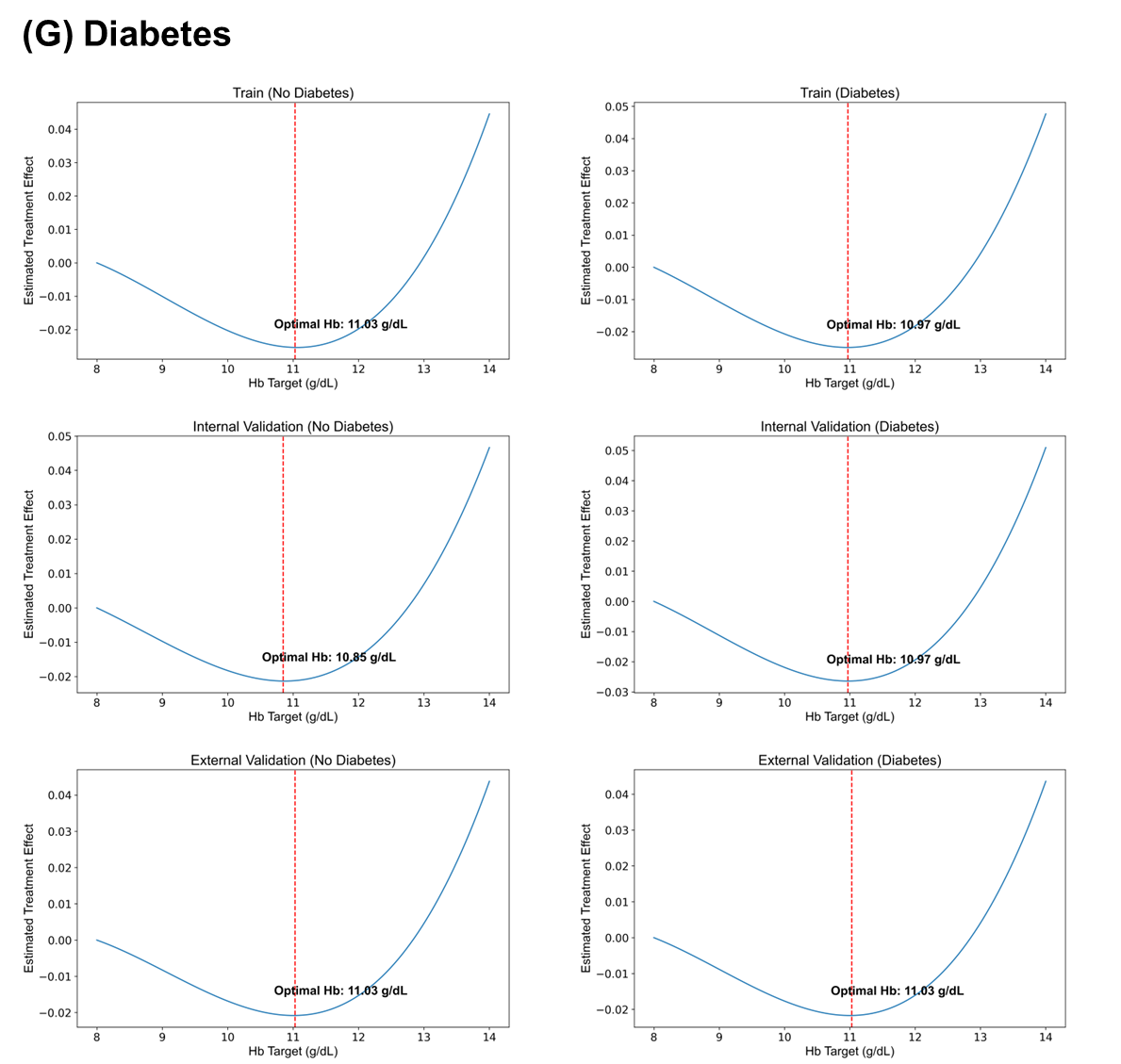


Supplementary Figure S5. Forest plot of multivariable logistic regression predicting individualized optimal hemoglobin targets below 10 g/dL. SBP, systolic blood pressure; DBP diastolic blood pressure; SpO₂, saturation of peripheral oxygen; FiO₂, fraction of inspired oxygen; RRT, renal replacement therapy; WBC, white blood cell; ALT, alanine aminotransferase; AST, aspartate aminotransferase; BUN, blood urea nitrogen; PT INR, prothrombin time international normalized ratio; aPTT, activated partial thromboplastin time; eGFR, estimated glomerular filtration rate; CKD, chronic kidney disease; ESKD, end-stage kidney disease.

(A) Train data


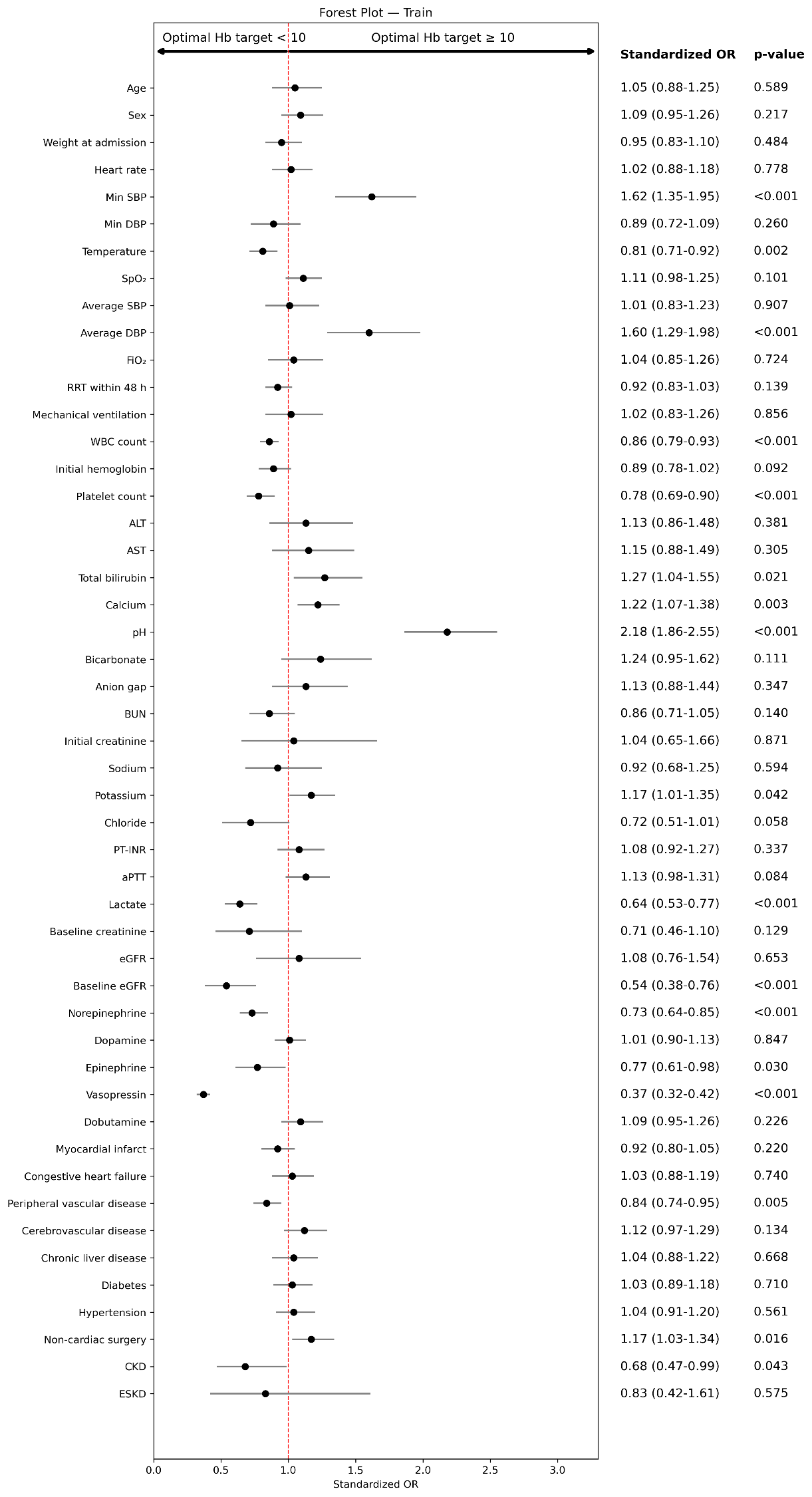


(B) Internal validation


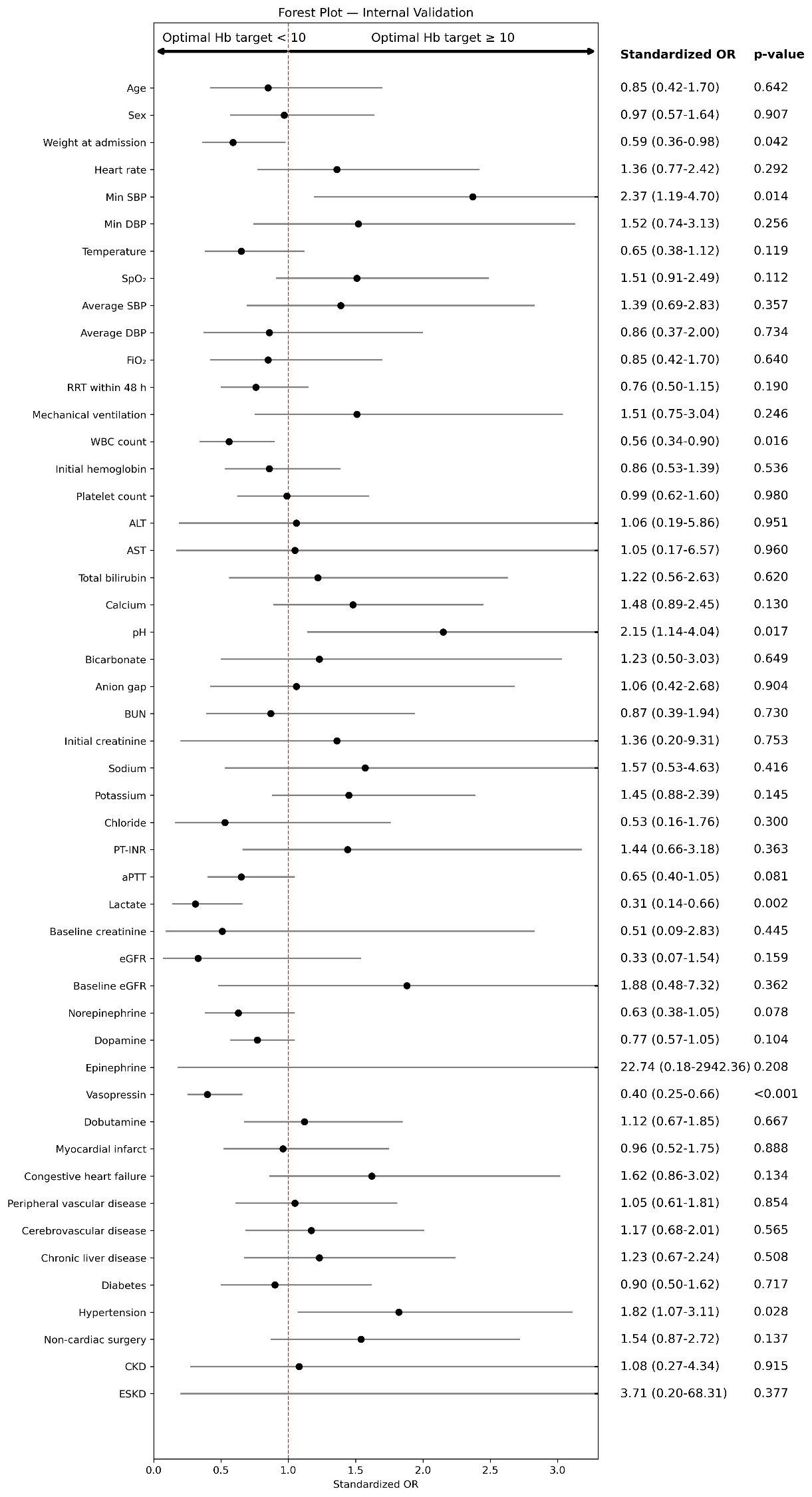


(C) External validation data


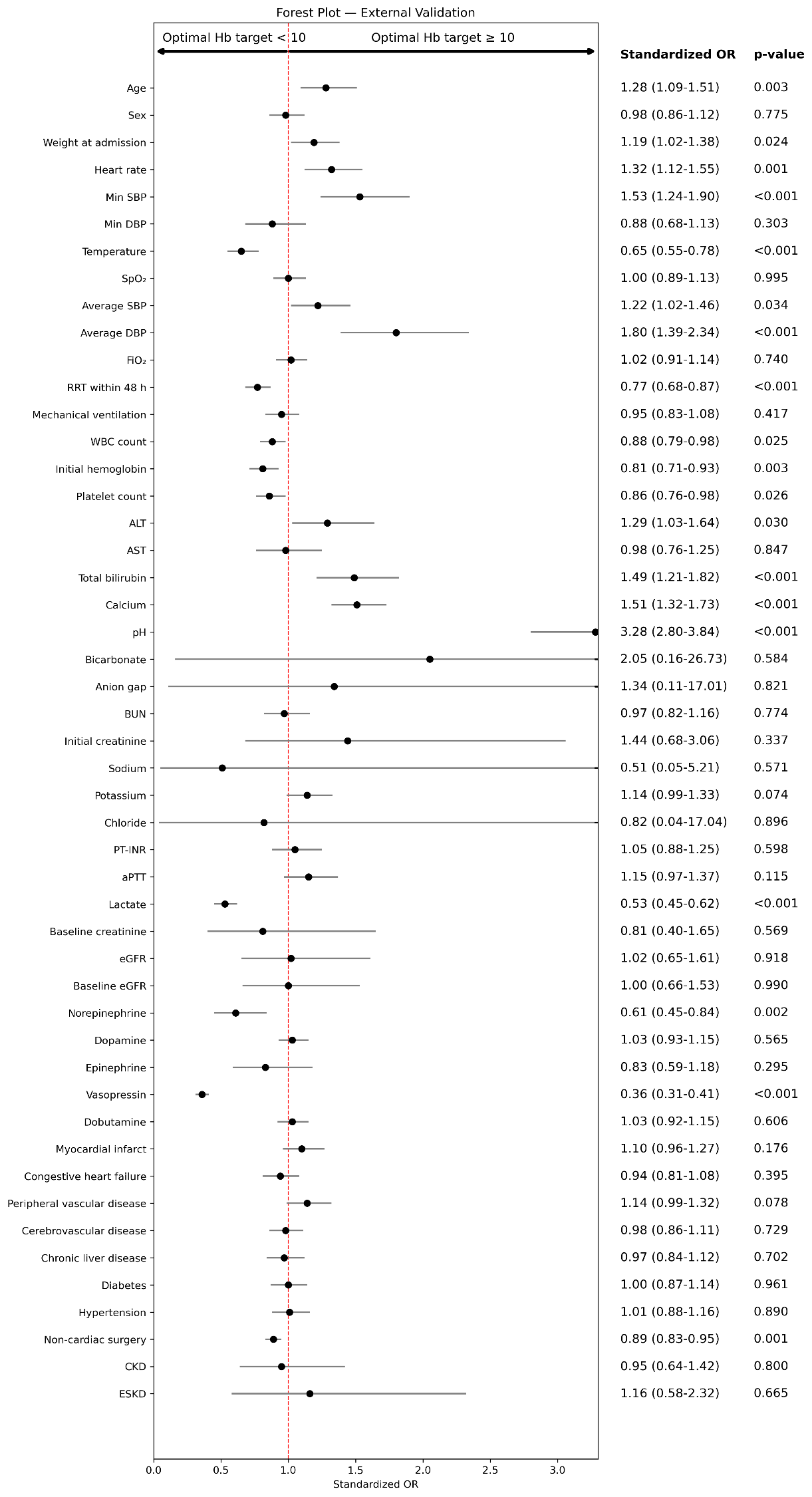

Supplement: Supplementary file 1 — Supplementary Material 1 [file 41598_2026_50363_MOESM1_ESM.docx]
